# Health disparities in transitions between kidney replacement therapy modalities and mortality in England: A multistate model using UK Renal Registry data

**DOI:** 10.1371/journal.pmed.1004674

**Published:** 2026-02-18

**Authors:** Jessica Potts, Camille M. Pearse, Mark Lambie, James Fotheringham, Harry Hill, David Coyle, Sarah Damery, Kerry Allen, Iestyn Williams, Simon J. Davies, Ivonne Solis-Trapala

**Affiliations:** 1 School of Medicine, Keele University, Keele, United Kingdom; 2 MRC Lifecourse Epidemiology Centre, University of Southampton, Southampton, United Kingdom; 3 Sheffield Centre for Health and Related Research, University of Sheffield, Sheffield, United Kingdom; 4 Patient Partnership Lead, NIHR Devices for Dignity MedTech Cooperative, Sheffield, United Kingdom; 5 Department of Applied Health Sciences, University of Birmingham, Birmingham, United Kingdom; 6 Health Services Management Centre, University of Birmingham, Birmingham, United Kingdom; University of Nottingham School of Medicine, UNITED KINGDOM OF GREAT BRITAIN AND NORTHERN IRELAND

## Abstract

**Background:**

While ethnic and deprivation-related disparities in kidney replacement therapy (KRT) initiation are well established, their impact on transitions between treatment modalities and mortality over the course of kidney failure remains poorly understood. This study aimed to examine the association between ethnicity and area-level deprivation and the rates of transition between treatment modalities and death across the patient life course on KRT.

**Methods and findings:**

We used a parametric multistate model to analyse UK Renal Registry data from 93,451 patients initiating KRT in England between 2005 and 2020 with a median follow-up of 1,497 days [IQR: 640−2,841] (4.1 years [IQR: 1.75,7.8]). We estimated transition-specific hazard rates and probabilities between peritoneal dialysis (PD), home haemodialysis (HHD), in-centre haemodialysis (ICHD), transplantation, and death using Weibull proportional hazard models. Ethnicity and area-level deprivation (measured by quintiles of the Index of Multiple Deprivation [IMD]) were included as covariates of primary interest, with models additionally adjusted for sex, age and diabetes mellitus as the primary kidney disease (PKD). Compared with White patients, Asian patients had lower transition rates from ICHD to PD (hazard ratio [HR]: 0.68, 95% confidence interval [CI] [0.51,0.91]), and from PD to ICHD (HR 0.85, 95% CI [0.78,0.92]), but a higher rate of returning to ICHD after transplantation (HR 1.12, 95% CI [1.01,1.24]). Black patients also had lower transition rates from ICHD to PD (HR 0.64, 95% CI [0.47,0.88]) and to HHD (HR 0.47, 95% CI [0.37,0.61]), but higher rates of transition from PD to ICHD (HR 1.16, 95% CI [1.01,1.33]) and from transplantation to ICHD (HR 1.73, 95% CI [1.44,2.08]). Patients living in the most deprived areas had lower transition rates from ICHD to PD (HR 0.63, 95% CI [0.56,0.70]), to HHD (HR 0.49, 95% CI [0.38,0.64]), and to transplantation (HR 0.57, 95% CI [0.52,0.64]), and higher rates from transplantation to ICHD (HR 1.63, 95% CI [1.43,1.85]) and to death (HR 1.53, 95% CI [1.33,1.76]), compared with those from the least deprived areas. A limitation of our study is that, apart from diabetes mellitus as the PKD, comorbidities were not included in the analysis due to incomplete reporting in the UK Renal Registry. This should be considered when interpreting the observed disparities, particularly those related to area-level deprivation.

**Conclusions:**

These findings highlight persistent inequalities throughout the KRT pathway. The multistate modelling framework applied in this study offers a foundation for future research to design and evaluate interventions that improve equity and patient outcomes in kidney care.

## Author summary

**Why was this study done?:**

- Kidney replacement therapy (KRT) options such as home dialysis and transplantation can improve quality of life, but not everyone has equal access to these treatments.
- Previous research has shown differences in who begins KRT, but much less is known about how these differences develop as patients move through different treatment stages over time.
- We wanted to examine how these disparities unfold over the full course of KRT by using national data and a statistical model that tracks how people move from one treatment to another over time.

**What did the researchers do and find?:**

- We analysed data from more than 93,000 adults in England who started KRT between 2005 and 2020. We estimated how people from different ethnic groups and living in areas with varying deprivation moved between treatment types or died.
- Asian and Black patients experienced less favourable transition patterns compared to White patients, with lower rates of moving to home therapies and higher rates of returning to hospital dialysis after a transplant.
- People living in the most deprived areas also faced barriers to accessing different treatment options. They had higher death rates and were more likely to return to in-centre haemodialysis.

**What do these findings mean?:**

- Inequalities appear throughout the KRT pathway and are associated with differences in who accesses home-based therapies or transplantation, and in who faces higher risks of death or returning to hospital-based treatment.
- The study identifies when and where these disparities occur, helping to identify critical points in the care pathway where equity-focussed interventions are most needed.
- The transition estimates produced by this study can support the design and evaluation of policy and service changes to improve outcomes and reduce disparities in kidney care.
- A limitation of the study is that information on health conditions other than diabetes (when diabetes was recorded as the main cause of kidney disease) was incomplete and therefore not included. This should be considered when interpreting the observed transition patterns, because having multiple health conditions may differ across groups and may affect treatment access and outcomes.

## Abstract 3

In an observational study, Jessica Potts and colleagues investigate health inequalities associated with the transitions in kidney therapy modality or death in the UK Renal Registry.Image: https://pixabay.com/illustrations/inequality-hierarchy-shadow-9923512/Credit: Pixabay AI generated

## Introduction

Chronic kidney disease (CKD) is a global public health priority and by 2040 is projected to be the fifth leading cause of death worldwide [1]. Kidney replacement therapy (KRT), which is lifelong for many patients, typically begins with dialysis, although some may receive a pre-emptive transplant, *i.e.*, a kidney transplant performed before dialysis becomes necessary. Transplantation is generally preferred due to its potential to improve quality of life compared to ongoing dialysis [2].

Dialysis treatment is burdensome and significantly impacts quality of life for patients with CKD [3]. Home-based dialysis, such as peritoneal dialysis (PD) or home haemodialysis (HHD), may be preferable for some patients [4], but access varies substantially. Evidence suggests that initiation of home dialysis is less likely among those from ethnic minoritised groups or from deprived communities [5,6]. Addressing these disparities is critical to improving outcomes and delivering equitable, patient-centred care.

Previous research [7] has largely focussed on access to home dialysis at treatment initiation and examined only the immediate next ‘event’, such as modality change, death, or transplantation. This approach overlooks how patients move between KRT modalities over time and how these transitions differ across patient groups. Traditional methods, such as Cox proportional hazards (PH) or competing risk models, typically focus on single endpoints and do not account for the sequence of intermediate events or interactions between different treatment states.

To address these gaps, this study aims to examine the life course of patients from the point of KRT initiation, through any changes in treatment modality (including PD, HHD, in-centre haemodialysis [ICHD], and transplantation), or death, and to identify health inequalities associated with these transitions. By providing a comprehensive analysis of modality transitions over time, this work aims to inform strategies to optimise treatment pathways and reduce inequities in KRT.

## Methods

### Ethics statement

We confirm that informed consent was not required for this study. The analysis used pseudonymised data obtained from the UK Renal Registry (UKRR), which is permitted to collect, process, and share confidential patient information for audit and research purposes under Section 251 of the NHS Act 2006. These permissions are reviewed and renewed annually by the Health Research Authority’s Confidentiality Advisory Group. The study was conducted under the ethical approvals granted by the UK Health Research Authority (Ref: 20-WA-0249) and by the Research Ethics Committee for the UKRR (Ref: 16/NE/0042). As all data were pseudonymised and collected under these existing approvals, individual informed consent was not required.

### Study population

Our study population included patients (>18 years) starting KRT in English renal centres between 1st January 2005 and 31st December 2020, identified and provided by the UK Renal Registry (UKRR). KRT modality was defined as either ICHD, HHD, PD, or transplantation. Information on patient characteristics was recorded at the time the patient initiated KRT, including sex, age, area-level deprivation, ethnicity and whether diabetes mellitus (DM) was the primary kidney disease (PKD). The date of death was provided by the UKRR through linkage with the Office for National Statistics.

### Measurement of area-level deprivation

Area-level deprivation was measured using population quintiles of the English Index of Multiple Deprivation (IMD), a composite measure of neighbourhood deprivation based on seven domains: income, employment, education, health, crime, barriers to housing and services, and the living environment. Quintile 5 represents the most deprived areas [8].

### Definition of patient ethnicity

Patient ethnicity was self-reported to centre staff and submitted to the UKRR by each dialysis centre. It was categorised in five groups based on the 2021 census [9]: Asian, Black, Mixed, White, and Other. Asian patients included those identifying as Bangladeshi, Pakistani, Indian, Chinese or “Other Asian”. Black patients included African, Caribbean and “Other Black”. Mixed patients were White and Asian, White and Black or “Other mixed”. White patients were British, Irish or “Other White”. Those identifying as Arab or “Any other ethnicity” were categorised as “Other”.

### Study design, aims and events of interest

This cohort study, using data from the UKRR, explored the life course of patients following KRT initiation, including transitions between treatment modalities and potential health inequalities, with a particular focus on disparities by ethnicity and area-level deprivation. Patients could experience transitions between five key treatment states: ICHD, HHD, PD, transplant, and death. Each observed transition from one state to another, including death, was considered an event of interest. Patients could enter the study from any treatment modality, allowing the capture of the timing and frequency of treatment changes and mortality throughout follow-up.

### Statistical analysis

Patient characteristics, including sex, IMD, ethnicity and DM as the PKD were summarised as frequencies (%), while age was presented as median (Interquartile range, [IQR]), both overall and by annual cohorts of KRT incident patients in England.

UKRR records on initial KRT modality, time to subsequent therapy changes, and mortality were used to fit a parametric multistate model, describing how individual patients transition between treatment modalities over their life course. The model comprised four transient states, ICHD, HHD, PD, and transplant, between which patients could transition, as well as an absorbing state of death.

**Fig 1:**
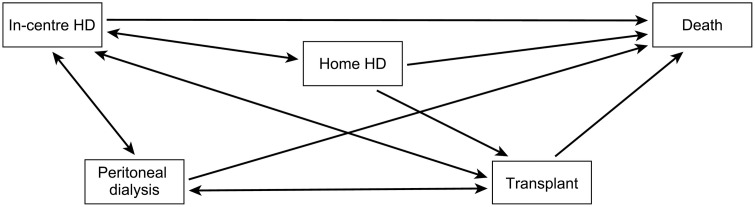
Diagram of the multistate model showing possible transitions between five states. Participants could enter the model from any treatment modality, and all allowed transitions are depicted. HD, haemodialysis.

Fig 1 illustrates the states and transitions of the multistate model, with arrows indicating possible transitions, and the permitted direction of movements within the model. Designed to reflect clinical practice and real-world evidence, the model incorporates three restrictions on transitions that are infrequently observed in practice and occur rarely in the dataset. Direct transitions between the two home dialysis modalities (HHD to PD (*n* = 32), PD to HHD (*n* = 68)), as well as from transplant to HHD therapy (*n* = 32), were excluded from the model, as they accounted for less than 1% of all observed transitions. This exclusion prevented issues with model parameter estimation due to sparse data.

Patients lost to follow-up for more than 120 days had their time to event censored at the point of loss. For follow-up gaps of less than 120 days, the previous state was carried forward until the next recorded state. A small subset of patients (2%) who, at some stage in their treatment trajectory, had the timeline code “recovered renal function” were censored at the last recorded treatment. For patients still alive and receiving treatment at the end of the data collection period, an administrative censoring date of 31st December 2020, was applied.

Transition-specific hazard functions were modelled using Weibull PH models. Ethnicity and IMD quintiles were included as covariates of primary interest, while models were additionally adjusted for age, sex, and a binary variable for DM as the PKD. DM was included as a covariate to account for its potential confounding effects on mortality, transplantation, and transitions between dialysis modalities. From these models, hazard ratios (HRs) and 95% confidence intervals (95% CIs) were estimated. Clinically relevant interactions, including those between ethnicity and IMD quintile, were screened; however, as no statistically significant interactions were identified, they were excluded from the final multistate model. Non-linearity in age was assessed by incorporating a quadratic term, but this effect was not statistically significant. To illustrate an ‘average’ effect, the characteristics of the typical study cohort participant, a White male, aged 62 years, with DM not as PKD, in the middle IMD quintile were selected. The median duration spent in each state before transitioning and the probability of state transitions at specific time points were estimated.

The transition-specific hazard functions were estimated separately using maximum likelihood assuming the semi-Markov property, where the probability of moving between states depends on the time elapsed since entering the current state. The R *flexsurv* package [10] was employed to fit the models. As this software does not support clustering, standard errors of the regression parameters were adjusted using inflation factors derived from accelerated failure time models that permitted clustering by centre, fitted with the R *survival* package [11]. This approach provides robust standard errors that reflect the correlation of outcomes within centres while keeping the model structure parsimonious. Model fit was assessed using cumulative hazard plots. To evaluate the adequacy of the Weibull PH specification, parameter estimates were additionally compared with those obtained from Cox PH models. An additional analysis was conducted to explore potential patterns in transition rates over time by fitting separate multistate models for the three patient cohorts who initiated treatment in 2005–2009, 2010–2014 and 2015–2020, respectively.

CIs were reported for all HRs but were not adjusted for multiple comparisons. As these analyses are exploratory, interpretation focuses on overall subgroup and temporal patterns rather than on individual statistically significant differences, which may arise by chance.

Missing data were limited (approximately 5% across variables). Analyses were therefore conducted using all available data. Although the extent of missingness was small, and substantial bias is therefore unlikely, the possibility of bias due to non-random missing data cannot be excluded. Statistical analysis was conducted using Stata Version 17 and R version 4.2.0.

This study was conducted according to the published “*Intervening to eliminate the centre-effect variation in home dialysis use (Inter-CEPt)*” protocol [12]. The original protocol included plans for multistate modelling of treatment modality history and mortality. During the course of the study, prior to data analysis, this analysis was further specified to explore whether ethnicity and area-level deprivation influenced treatment transitions. The adjustment for DM as PKD was not pre-specified but was introduced at the analysis stage in response to reviewer feedback, as it was considered a potential confounder.

This study is reported as per the Reporting of Studies Conducted using Observational Routinely-Collected Data (RECORD) guideline, S1 RECORD Checklist.

## Results

### Study cohort

93,451 people initiated KRT between 2005 and 2,020 in 51 English dialysis and transplant centres, generating 215,036 timeline codes indicating the patient status, ranging from 1 to 49 per patient (median 2 IQR [1,2]) with median follow-up times of 1,497 days (IQR [640,2,841]). 2,945 (3%) patients were lost to follow-up for more than 120 days and 1,759 (2%) patients had a timeline code “recovered renal function”; 50,231 (54%) patients were still receiving KRT at the end of the follow-up period (S1 Fig). Data were right-censored at the date of last known follow-up. For patients with censored records, all treatment episodes prior to censoring were retained, and no participants were excluded from the dataset.

Over the 15-year period, the proportions of incident patients receiving ICHD, HHD or PD as starting modality were stable (S2 Fig). ICHD was the initial KRT for 73% of patients, (*n* = 67,910), whereas between 2005 and 2013, patients receiving pre-emptive transplant increased by approximately 6% (from 3% to 9%). This remained stable from 2013 onwards until 2020 where a small reduction was seen, coincident with the start of the COVID-19 pandemic. Initial KRT modality was a home dialysis in 20% of patients. The demographics of incident KRT patients each year are shown in S1 Table. Across the 15-year period, there was consistency in the age, sex, ethnicity, IMD quintile and DM as KPD distribution. Therefore, the demographics of incident KRT patients were collapsed and stratified by the initial KRT treatment (Table 1). S3 Fig demonstrates the intersectionality of area-level deprivation and ethnicity with minoritised ethnic groups over-represented in the lower IMD quintiles.

**Table 1:** Initial KRT modality, stratified by age, sex, ethnicity and IMD quintile, and diabetes mellitus as PKD.

	ICHD	HHD	PD	Transplantation	Overall
**Patients (*N*, %)**	67,894 (73%)	144 (<1%)	18,906 (20%)	6,507 (7%)	93,451
**Age (Median, IQR)**	67 (54,76)	61 (50,69.5)	60 (47,72)	49 (39,59)	64 (51,75)
**Sex: Male (*N*, %)**	43,166 (64%)	94 (65%)	11,882 (63%)	3,818 (59%)	58,960 (63%)
**Sex: Female (*N*, %)**	24,728 (36%)	50 (35%)	7,024 (37%)	2,689 (41%)	34,491 (37%)
**Ethnicity (*N*, %)**					
**Asian**	8,603 (13%)	12 (8%)	2,349 (12%)	641 (10%)	11,605 (12%)
**Black**	5,279 (8%)	4 (3%)	1,220 (6%)	188 (3%)	6,691 (7%)
**Mixed**	837 (1%)	1 (1%)	238 (1%)	103 (2%)	1,179 (1%)
**Other**	1,026 (2%)	0 (0%)	260 (1%)	80 (1%)	1,366(2%)
**White**	48,403 (71%)	125 (87%)	14,286 (76%)	5,350 (82%)	68,164 (73%)
**Missing**	3,756 (5%)	2 (1%)	553 (3%)	145 (2%)	4,446 (5%)
**IMD quintile (*N*, %)**					
**1 (Least deprived)**	9,381 (14%)	22 (15%)	3,287 (17.4%)	1,451 (22%)	14,141 (15%)
**2**	11,469 (17%)	27 (19%)	3,702 (19.6%)	1,371 (21%)	16,539 (18%)
**3**	13,296 (20%)	30 (21%)	3,835 (20.3%)	1,360 (21%)	18,521 (20%)
**4**	15,843 (23%)	32 (22%)	4,076 (21.5%)	1,211 (19%)	21,12 (23%)
**5 (Most deprived)**	17,829 (26%)	33 (23%)	3,992 (21.1%)	1,055 (16%)	22,909 (25%)
**Missing**	106 (<1%)	0 (0%)	14 (<1%)	59 (1%)	179 (<1%)
**DM as PKD: No (*N*,%)**	46,478 (68%)	102 (71%)	13,680 (72%)	5.339 (82%)	65,599 (70%)
**Yes (*N*,%)**	17,466 (26%)	38 (26%)	4,638 (25%)	888 (14%)	23,031 (25%)
**Missing**	3,950 (6%)	4 (3%)	588 (3%)	280 (4%)	4,822 (5%)

IMD, Index of Multiple Deprivation; DM, diabetes mellitus; PKD, primary kidney disease; KRT, kidney replacement therapy; ICHD, in-centre haemodialysis; HHD, home haemodialysis; PD, peritoneal dialysis

Transitions between modalities are illustrated in a Sankey diagram (S4 Fig), which provides a visual summary of patient flows corresponding to the detailed counts in S2 Table. S2 Table summarises the frequency of initial and subsequent state of all observed transitions, either to another therapy modality or to death, for the 65,172 (70%) patients who experienced at least one transition during the study follow-up. Among all transitions from PD, 56% switched to ICHD; for those transitioning from HHD, 63% switched to ICHD; and 53% of post-transplant transitions also led to ICHD. For patients on ICHD, the most frequent transition was to death, accounting for 59% of these cases. Similar proportions of patients across the three dialysis modalities eventually received a transplant. The estimated median time spent in each state, including those who remained on a single modality, is illustrated in S3 Table.

Table 2 presents HRs and 95% CIs for transitions between treatment states, estimated from multivariable models, with ethnicity and IMD as the covariates of primary interest and adjusted for sex, age and DM as the PKD.

**Table 2:** Hazard ratios (95% CI) for transitions from ICHD, PD, HHD and transplant to each of the other states.

	Transition from ICHD to:				Transition from PD to:			Transition from HHD to:			Transition from transplant to:		
	PD	HHD	Transplant	Death	ICHD	Transplant	Death	ICHD	Transplant	Death	ICHD	PD	Death
**Ethnicity**													
***Asian***	0.68 (0.51,0.91)	0.31 (0.24,0.41)	1.14 (1.03,1.27)	0.65 (0.62,0.69)	0.85 (0.78,0.92)	0.93 (0.86,1.02)	0.82 (0.73,0.92)	0.81 (0.59,1.10)	0.87 (0.57,1.33)	0.65 (0.39,1.10)	1.12 (1.01,1.24)	0.80 (0.51,1.27)	0.90 (0.77,1.04)
***Black***	0.64 (0.47,0.88)	0.47 (0.37,0.61)	0.88 (0.79,0.97)	0.54 (0.51,0.57)	1.16 (1.01,1.33)	0.68 (0.58,0.79)	0.55 (0.43,0.70)	0.86 (0.58,1.27)	1.04 (0.73,1.49)	0.37 (0.19,0.71)	1.73 (1.44,2.08)	1.03 (0.66,1.59)	0.86 (0.70,1.05)
***Mixed***	0.74 (0.60,0.90)	0.59 (0.34,1.02)	1.03 (0.81,1.30)	0.62 (0.54,0.70)	1.09 (0.81,1.46)	0.90 (0.67,1.20)	0.74 (0.59,0.92)	0.81 (0.34,1.94)	1.06 (0.62,1.83)	1.08 (0.34,3.42)	1.20 (0.81,1.77)	0.45 (0.14,1.39)	0.84 (0.58,1.24)
***Other***	0.59 (0.36,0.98)	0.32 (0.17,0.60)	0.96 (0.83,1.11)	0.56 (0.50,0.61)	0.96 (0.81,1.14)	0.86 (0.62,1.18)	0.61 (0.45,0.83)	0.83 (0.45,1.53)	0.82 (0.34,1.97)	0.66 (0.15,2.89)	1.03 (0.72,1.45)	0.51 (0.18,1.48)	0.69 (0.50,0.97)
***White***	Reference	Reference	Reference	Reference	Reference	Reference	Reference	Reference	Reference	Reference	Reference	Reference	Reference
**IMD quintile**													
***(Least deprived) 1***	Reference	Reference	Reference	Reference	Reference	Reference	Reference	Reference	Reference	Reference	Reference	Reference	Reference
***2***	0.91 (0.84,0.98)	0.94 (0.81,1.09)	0.91 (0.84,0.97)	1.04 (1.00,1.08)	1.03 (0.98,1.10)	0.87 (0.79,0.94)	1.01 (0.92,1.10)	1.01 (0.82,1.23)	0.88 (0.73,1.06)	1.02 (0.75,1.38)	1.20 (1.05,1.38)	0.97 (0.70,1.36)	1.22 (1.06,1.41)
***3***	0.82 (0.73,0.92)	0.70 (0.57,0.85)	0.83 (0.79,0.88)	1.01 (0.96,1.06)	0.99 (0.93,1.06)	0.78 (0.70,0.88)	1.00 (0.92,1.10)	0.80 (0.64,1.02)	0.76 (0.60,0.95)	1.04 (0.71,1.53)	1.31 (1.13,1.53)	1.18 (0.85,1.65)	1.20 (1.04,1.38)
***4***	0.72 (0.62,0.83)	0.62 (0.50,0.77)	0.72 (0.66,0.79)	1.05 (1.00,1.10)	1.03 (0.97,1.11)	0.71 (0.63,0.81)	1.07 (0.97,1.17)	0.87 (0.65,1.16)	0.67 (0.52,0.87)	1.33 (0.88,2.00)	1.47 (1.32,1.63)	0.78 (0.58,1.06)	1.40 (1.22,1.61)
***(Most deprived) 5***	0.63 (0.56,0.70)	0.49 (0.38,0.64)	0.57 (0.52,0.64)	1.08 (1.03,1.13)	1.05 (0.97,1.14)	0.55 (0.48,0.63)	1.23 (1.11,1.36)	0.82 (0.60,1.12)	0.64 (0.50,0.83)	1.33 (0.95,1.88)	1.63 (1.43,1.85)	0.96 (0.68,1.36)	1.53 (1.33,1.76)
**Sex**													
***Male***	Reference	Reference	Reference	Reference	Reference	Reference	Reference	Reference	Reference	Reference	Reference	Reference	Reference
***Female***	0.96 (0.87,1.07)	0.89	0.85 (0.81,0.89)	0.96 (0.94,0.99)	0.86 (0.82,0.90)	0.89 (0.83,0.95)	1.00 (0.94,1.06)	1.03 (0.89,1.20)	0.80 (0.66,0.96)	1.08 (0.91,1.28)	1.00 (0.94,1.06)	1.44 (1.23,1.68)	0.88 (0.80,0.96)
		(0.80,0.98)											
**Diabetes mellitus as PKD**													
**No**	Reference	Reference	Reference	Reference	Reference	Reference	Reference	Reference	Reference	Reference	Reference	Reference	Reference
**Yes**	0.92 (0.84,1.01)	0.77 (0.68,0.87)	0.76 (0.70,0.82)	1.30 (1.26,1.34)	1.18 (1.13,1.23)	0.71 (0.65,0.77)	1.81 (1.67,1.96)	1.18 (1.00,1.40)	0.81 (0.64,1.04)	1.85 (1.50,2.29)	1.23 (1.12,1.35)	0.51 (0.37,0.71)	1.86 (1.68,2.07)
**Age (per year)**	0.974 (0.972,0.975)	0.959 (0.957,0.961)	0.947 (0.946,0.948)	1.030 (1.029,1.031)	0.993 (0.992,0.994)	0.952 (0.951,0.954)	1.050 (1.048,1.051)	1.017 (1.004,1.010)	0.974 (0.69,0.979)	1.040 (1.020,1.050)	0.995 (0.992,0.997)	0.983 (0.976,0.990)	1.070 (1.060,1.070)

Multistate model results showing hazard ratios and 95% confidence intervals for transitions between treatment states. All estimates are from multivariable models, with ethnicity and IMD as the covariates of primary interest, adjusted for sex, age, and diabetes mellitus as PKD. Confidence intervals are not adjusted for multiple comparisons; interpretation should focus on overall patterns rather than isolated significant findings. IMD, Index of Multiple Deprivation; PKD, primary kidney disease; ICHD, in-centre haemodialysis; HHD, home haemodialysis; PD, peritoneal dialysis.

### Rates of transitions from ICHD

For patients receiving ICHD there is inequity in transition to home dialysis and transplantation by ethnicity and IMD, (Table 2), with those of Asian, Black, Mixed and ‘Other’ ethnicity showing lower hazard rates of transition to either PD or HHD compared to the White group. Those in the Asian group have a 14% higher hazard rate (HR 1.14, 95% CI [1.03,1.27]) of transplantation than White patients, whereas in Black patients this is 13% lower (HR 0.88, 95% CI [0.79,0.97]). Controlling for ethnicity and other patient-level factors, for each increasing IMD quintile there is a graded relative decrease in transitions rates to PD, HHD and transplantation but an increase in the hazard of death. A similar pattern is seen for increasing age, whereas female patients have a lower hazard of death or transition to transplant or HHD compared to male patients. Those with DM as PKD have lower rates of transition to home dialysis and transplantation, and higher rates of death, compared with those for whom DM was not the PKD.

### Rates of transition from PD

Asian patients on PD have a lower hazard rate of transitioning to ICHD, whereas in Black patients the transition hazard is higher (Table 2). Both Asian and Black patients on PD have a lower rate of transplantation (7% (HR 0.93, 95% CI [0.86,1.02]) and 32% (HR 0.68, 95% CI [0.58,0.79]) respectively) compared to White patients. Black and ‘Other’ ethnicities have a lower hazard of death compared to White patients.

IMD was not associated with transition rates to ICHD or death except in the most deprived area where transition rates to ICHD and death were higher. Transition rates to transplantation were lower for areas of higher deprivation. Females have lower rates of transition to ICHD (14% (HR 0.86, 95% CI [0.82,0.90])) and transplant (11% (HR 0.89, 95% CI [0.83,0.95])) compared to males. Each 10-year difference in age was associated with an increase in the hazard rate to death (HR 1.24, 95% CI [1.23,1.25] per 10 years) and a decrease in the hazard rate for transitions to ICHD (HR 0.97, 95% CI [0.96,0.98] per 10 years) and transplant (HR 0.81 [0.80,0.82] per 10 year). Patients with DM as PKD have an 80% (HR 1.81, [1.67,1.96]) increase in the hazard rate for transitions to death, lower rates of transplantation (HR 0.71, 95% CI [0.65,0.77] and higher rates of transition to ICHD (HR 1.18, 95% CI [1.3,1.23]) compared to those without.

### Rates of transition from HHD

Due to the relatively small number of patients using HHD, the CIs for the transition HRs are wide, and no clear patterns are observed for most transitions (Table 2). However, individuals living in neighbourhoods with higher deprivation have a lower hazard of transitioning to ICHD or transplantation compared to those in less deprived areas. Compared to White patients, Black patients on HHD have a lower risk of death, and among those on HHD, females have a lower transition rate to transplantation.

### Rates of transition from transplant

Asian and Black patients with a transplant had higher rates of transition to ICHD compared to White patients (12% (HR 1.12, 95% CI [1.01,1.24]) and 73% (HR 1.73, 95% CI [1.44,2.08]) respectively), (Table 2). Increasing IMD quintile was associated with an incremental hazard rate of death and transition to ICHD. Females had lower rates of death than males and higher rates of transition to PD. Age and DM as PKD had the expected associations with the hazard of death.

### Probability of transition over a 5-year period

**Fig 2:**
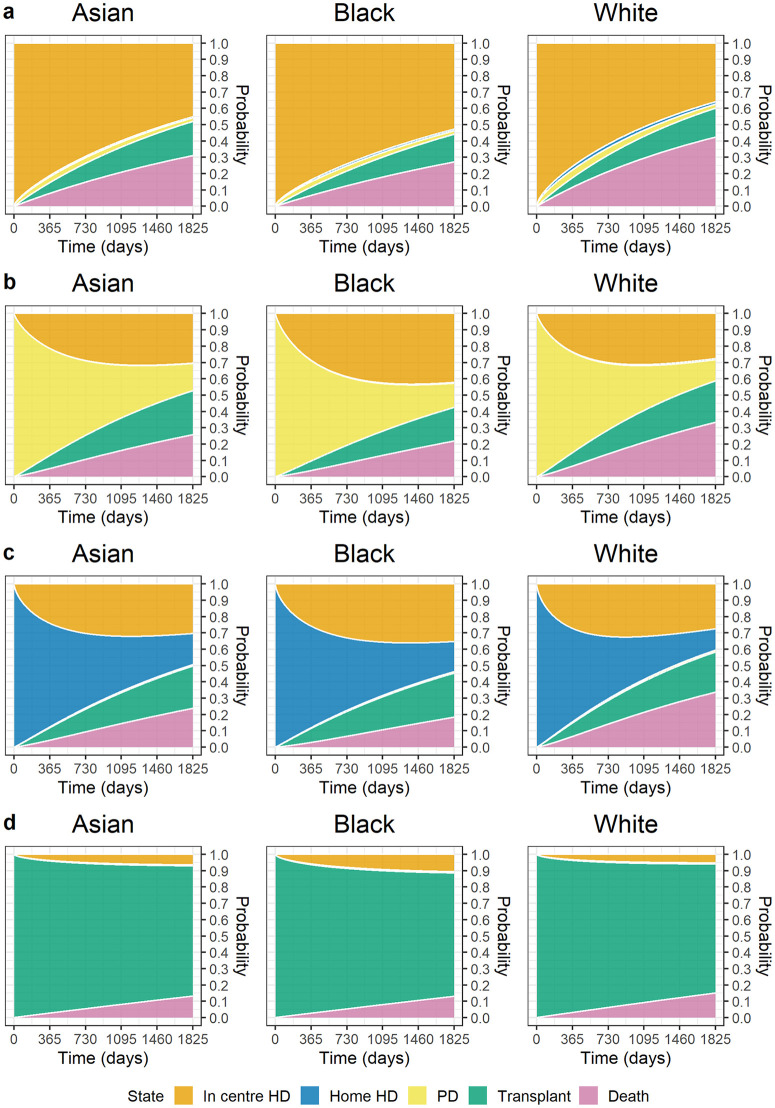
Estimated probability of transition for an Asian, Black, or White male aged 62 without diabetes mellitus as primary kidney disease in IMD quintile 3 (moderate deprivation level), commencing KRT on (a) ICHD, (b) PD, (c) HHD or (d) transplant. At each time point the probability of transition was estimated and graphs were created to show, over a 5-year period, the probability of transitioning into a new state for different ethnic groups, holding other characteristics fixed. IMD, Index of Multiple Deprivation; KRT, kidney replacement therapy; ICHD, in-centre haemodialysis; HHD, home haemodialysis; PD, peritoneal dialysis; HD, haemodialysis.

**Fig 3:**
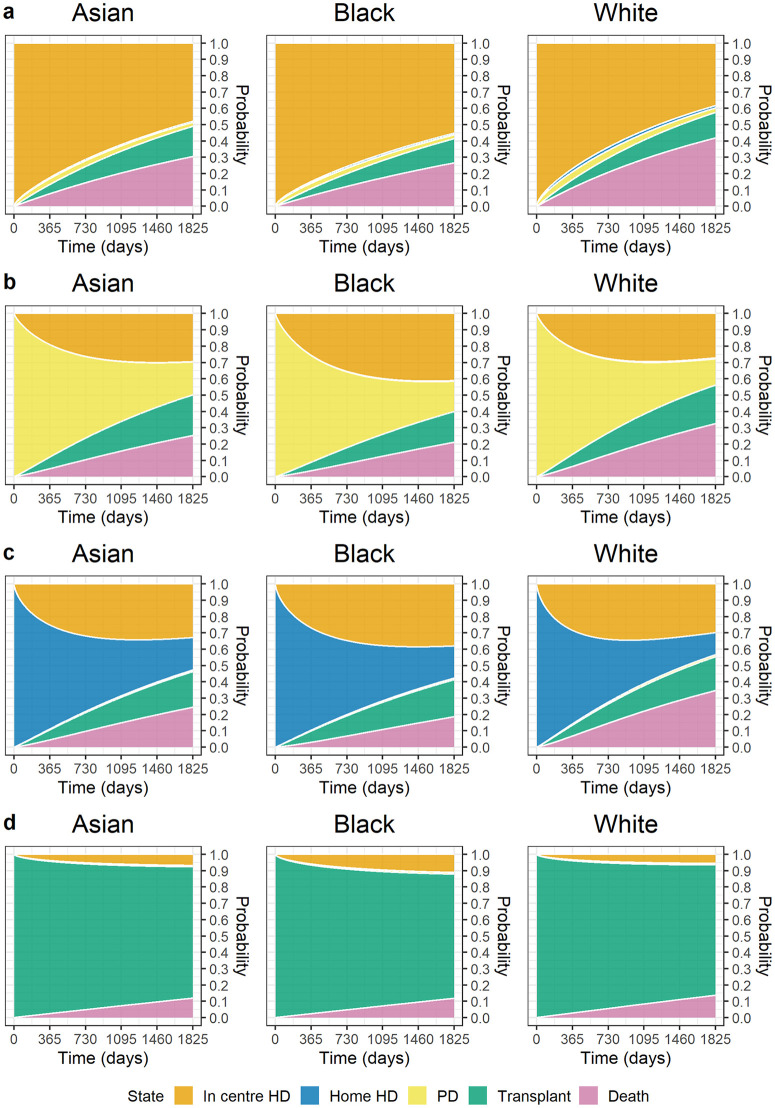
Estimated probability of transition for an Asian, Black, or White female aged 62 without diabetes mellitus as primary kidney disease in IMD quintile 3 (moderate deprivation level), commencing KRT on (a) ICHD, (b) PD, (c) HHD or (d) Transplant. At each time point the probability of transition was estimated and graphs were created to show, over a 5-year period, the probability of transitioning into a new state for different ethnic groups, holding other characteristics fixed. IMD, Index of Multiple Deprivation; KRT, kidney replacement therapy; ICHD, in-centre haemodialysis; HHD, home haemodialysis; PD, peritoneal dialysis; HD, haemodialysis.

Fig 2 illustrates the probability of transitioning between treatment modalities and mortality during 5 years from KRT start by ethnicity (Asian, Black and White ethnicities) for the average individual characteristics (62-year-old male, IMD quintile 3, DM was not PKD) according to KRT initial modality. Similar graphs for a typical female are presented in Fig 3. The corresponding figures for the Mixed and Other ethnicities are presented in S5 and S6 Figs in the Supporting information for males and females, respectively.

### Temporal patterns in transition rates

Figs A–K in S1 File illustrate potential temporal patterns across 3 different time periods: for Asian versus White patients, there were lower PD-to-ICHD rates, slightly higher PD-to-transplant rates, and lower transplant-to-PD transition rates over time (Fig A S1 File); as well as higher post-transplant mortality in more deprived areas (Figs E–H S1 File). Over time decreasing rates of transplant-to-PD transitions were observed among females (Fig I S1 File); and a decreasing rate of HHD-to-death transitions for older patients (Fig J S1 File). Patients with DM as PKD have increasing ICHD to HHD transition rates over time (Fig K S1 File).

## Discussion

As part of the inter-CEPt project [12], this study used multistate modelling of a large, nationally representative cohort of incident kidney failure cases spanning 15 years to examine how health inequalities by ethnicity and area-level deprivation influence patient outcomes [5,6]. These disparities are evident not only at treatment initiation but also across subsequent transitions between treatment modalities. By identifying where inequities persist throughout the treatment journey, our findings provide a foundation to inform interventions aimed at improving equitable access to home dialysis and other kidney replacement therapies.

Multistate models have previously been applied to transitions between health states in kidney failure, including dialysis access [13,14], hospitalisations by treatment modality [15], cardiovascular mortality risk [16], burden of delayed graft function [17], and clinical decision-making [18,19]. They have also been used to illustrate methodological developments [20]. This study provides a large-scale national analysis of transitions across all major kidney failure treatment modalities within a single multistate framework, enabling simultaneous, time-to-event analysis across the entire KRT pathway. This approach estimates transition-specific hazard rates in continuous time, stratified by patient characteristics, and provides a dynamic view of treatment trajectories. It offers valuable insight into persistent health inequalities that extend beyond initial treatment access.

By quantifying the time patients spend in each treatment modality and estimating transition probabilities over time, this model provides flexible and clinically relevant prognostic information for individuals with kidney failure. Its longitudinal, system-level perspective also highlights key transition points in the treatment pathway where disparities are most pronounced, offering crucial parameters to guide the design and evaluation of future interventions aimed at improving equity and outcomes.

Our findings indicate that the initial KRT modality in England remained relatively stable over a 15-year period. Annually, approximately 70% of patients start KRT on ICHD, 20% on PD and less than 1% on HHD, although it should be recognised that the latter requires a considerable training period that is often initially coded as ICHD. Between 2005 and 2013, there was a modest increase in the proportion of patients undergoing a pre-emptive transplant which does not appear to have had a notable impact on home dialysis use. This may reflect efforts to increase access to home dialysis, as seen in the shifting age profile of PD patients, with a higher proportion over 70, likely due to the increased use of assisted PD, i.e., the use of a paid healthcare assistant to help set up and deliver the dialysis treatment in the home [21].

Beyond this, little change was observed in patient demographics over the 15-year follow-up. Notably, worsening area-level deprivation is linked to a higher representation of individuals requiring KRT, reflecting the fact that CKD disproportionately affects more disadvantaged people [22,23]. There is a modest increase in the proportion of Asian patients starting KRT over time (14% in 2020 versus 10% in 2010).

Our longitudinal analysis aimed to determine whether previously observed disparities in access to home dialysis also influenced subsequent transitions between modalities during the life-course of kidney failure treatment. Our findings suggest that this is the case, both when switching from ICHD and after returning to dialysis following transplant failure. These disparities are particularly pronounced among underrepresented ethnic groups, especially Black patients, and are more substantial for HHD than PD.

Intersectionality between ethnicity and area deprivation plays an important role given the high dependency HHD in particular has on adequate home space and family support [24]. However, our research points to additional factors, including important centre factors such as institutional culture that affects the perception of eligibility by staff for home dialysis, engagement with quality improvement, staffing levels and patient trust in the institution [25,26]. While area-level deprivation is linked to lower access to home dialysis, it does not significantly impact the retention on this modality, except for the highest area deprivation quintile. For those on PD, higher deprivation increases the risk of switching to ICHD or death, whereas for HHD, the risk of these two outcomes may compete, resulting in a lower risk of switching to ICHD but a heightened risk of death. Although White patients were generally from less deprived areas, they exhibited higher mortality, consistent with prior studies [27] showing a relative survival disadvantage compared to ethnic minority groups. The mechanisms underlying this pattern remain unclear and may include unmeasured comorbidity or selection factors, highlighting the complexity of interpreting ethnicity-related outcomes beyond deprivation alone. Similarly, when interpreting modality transitions, our multistate model, which accounts for death as a competing risk, suggests that lower transition rates to PD, HHD, or transplantation in non-white ethnic groups likely reflect differences in modality access, although residual confounding cannot be excluded.

Limited access to transplantation among Black patients or those living in a neighbourhood of high deprivation level is well-documented. This is partly due to lower rates of transplant waitlisting and/or consideration for live donation [28,29]. In contrast, Asian patients have the highest transplant access, likely reflecting a concerted effort to improve waitlisting [30]. As in the US, the outcome of transplantation in Black people when compared to White people is worse as evidenced by the much higher risk of transfer to ICHD, whereas risk of death was consistently lower in this ethnic group [31].

As shown in Table 1, pre-emptively transplanted patients were younger and from less deprived areas, factors that likely influence both subsequent transitions and outcomes. These baseline differences should be considered when interpreting the higher transplant success and lower mortality observed in this group. Future studies could further stratify kidney transplant recipients by donor type to better evaluate potential disparities in living donor transplantation.

The median time spent on home dialysis modalities before transplantation (PD and HHD 1.8 years) or death (PD 2.1, HHD 2.3 years) was substantially shorter than on ICHD (2.6 and 3.1 years respectively). Time spent on PD in the UK when compared to other countries participating in the Peritoneal Dialysis Outcomes and Practice Patterns Study was relatively short due to notably earlier transplantation and this likely also explains the relatively short period on HHD [32].

Consistent with previous reports [33], the survival advantage of females in the general population is almost absent during dialysis [7], and mostly restored in the transplant population [33]. Despite a higher proportion of females receiving pre-emptive transplantation, females are less likely to receive a transplant once dialysis has commenced. Strategies to reduce sex-based disparities in transplantation access have recently been proposed in the US [34].

Our findings also highlight modest potential temporal patterns in treatment transitions. Over time, the rate of females moving from transplantation to PD has declined, while older patients have experienced a decreasing rate in HHD-to-death transitions. Among Asian patients compared to White patients, there are lower rates of switching from PD to ICHD, a modest increase in PD-to-transplant transitions, and reduced transitions from transplantation back to PD. Additionally, post-transplant survival has improved in more deprived areas, underscoring the evolving landscape of kidney failure treatment and its intersection with sociodemographic factors. As this analysis was exploratory and CIs were not adjusted for multiple comparisons, isolated statistically significant findings should be interpreted with caution, with emphasis placed on overall patterns across transitions.

A major strength of this study is the application of a multistate model to a large UK Renal Registry cohort, enabling the analysis of transitions between KRT modalities throughout the patient life course. This approach provides a dynamic and comprehensive view of treatment trajectories, capturing the timing and sequence of modality changes, and offering insights into patient experiences and outcomes. The large, population-based registry of patients initiating KRT in England, long-term follow-up and minimal missing data (<5%) minimises selection bias and enhances generalisability. Analyses were adjusted for key factors, including age, sex and DM as the PKD, reducing confounding in transitions between treatment modalities and outcomes.

There are several limitations. Our multistate model can handle both intermittent missing data and right censoring under the assumption that they are unrelated to the missing data given the observed data. However, this assumption cannot be verified. To specify a parsimonious model that reflected real-world practice some transitions were not allowed in the model, notably switches from PD to HHD and vice-versa. An additional limitation is that the exploratory analysis of transition rates over time was performed by fitting three separate multistate models for distinct cohorts, rather than including the KRT start period as a time-varying covariate within the single full-cohort multistate model. This more complex approach is currently unsupported by available statistical software. Our study was explorative and observational, based on data collected by the UKRR. We were not able to adjust our analyses for comorbidities, apart from DM as the PKD, as this information was incompletely reported by centres to the registry over the study period. Multimorbidity is strongly associated with income, educational attainment, and area-level deprivation in the UK, and it is therefore likely that it plays an important part in access to home dialysis and transplantation [35]. A notable proportion of patients in our cohort (12%) are of South Asian ethnicity, reflecting the UK’s demographic composition [9], particularly the Indian, Pakistani and Bangladeshi communities. This may limit the generalisability of our findings to countries with different ethnic distributions. Another limitation of our study is that the UK Renal Registry does not capture the reasons for KRT modality transitions. While many transitions are clinically driven, it remains possible that the disparities we observed are related, at least in part, to underlying health inequalities.

Our life-course modelling of KRT use reveals persistent structural inequalities in access, treatment transitions, and outcomes for people with kidney failure in England. Underrepresented ethnic groups and individuals from deprived areas face disadvantages. Tackling these disparities requires coordinated interventions across the entire treatment pathway, alongside broader efforts to address the social determinants of health. The multistate modelling framework presented in this study offers a powerful tool to inform the design and evaluation of policies and service delivery changes aimed at improving care pathways and advancing health equity. Building on the inequalities highlighted by the multistate modelling, graphical modelling [26] mapped the complex relationships between patient- and centre-level factors associated with home dialysis uptake. Together, these modelling approaches provided a rigorous, data-driven foundation for the LOCAL (Location of Dialysis Care in Kidney Life) intervention [36], which targets modifiable centre-level determinants through leadership development, organisational culture change, adequate staffing and resourcing, quality improvement initiatives, and strategies to support patient empowerment, including assisted PD.

## Supporting information

S1 Table Initial KRT modality, sex, IMD quintile, ethnicity, diabetes mellitus as primary kidney disease (*N* (%) within each stratum across years) and age (median, IQR) of KRT incident patients. KRT: Kidney replacement therapy, IMD, Index of Multiple Deprivation; DM, diabetes mellitus; PKD, primary kidney disease; ICHD, in-centre haemodialysis; HHD, home haemodialysis; PD, peritoneal dialysis.
(DOCX)

S2 Table Frequency of transition between treatment modalities ^a^ or to death, with previous modality cross-tabulated against current modality at successive observation times. ^a^ There are no restrictions in the number of modality changes a patient can undergo; therefore, patients may experience multiple modality transition pairings. ^b^ Transition not included in the multistate model due to very low frequency. ICHD, in-centre haemodialysis; HHD, home haemodialysis; PD, peritoneal dialysis.
(DOCX)

S3 Table Estimated median time spent in each state, in days (inter-quartile range), before transitioning to the next state^a^ and overall median time spent in same modality. ^a^ Estimated for a White male aged 62 with diabetes mellitus not as primary kidney disease in IMD quintile 3 (moderate area-level deprivation). ICHD, in-centre haemodialysis; HHD, home haemodialysis; PD, peritoneal dialysis; IMD, Index of Multiple Deprivation.
(DOCX)

S1 Fig Flow of participants and observations through data cleaning. KRT, kidney replacement therapy.
(TIF)

S2 Fig Proportion of patients on each starting treatment for incident KRT patients. KRT, kidney replacement therapy; HD, haemodialysis.
(TIF)

S3 Fig Intersectionality of area-level deprivation and ethnicity, illustrated by the proportion of the ethnic composition within each IMD quintile. IMD, Index of Multiple Deprivation.
(TIF)

S4 Fig Sankey diagram showing the start and end states of all consecutive transitions. The width of each flow is proportional to the number of patients transitioning between states. ICHD, in-centre haemodialysis; HHD, home haemodialysis; PD, peritoneal dialysis.
(TIFF)

S5 Fig Estimated probability of transition for a Mixed or Other male aged 62 without diabetes mellitus as primary kidney disease in IMD quintile 3 (moderate deprivation level), commencing KRT on (a) ICHD, (b) PD, (c) HHD or (d) Transplant. IMD, Index of Multiple Deprivation; KRT, kidney replacement therapy; ICHD, in-centre haemodialysis; HHD, home haemodialysis; PD, peritoneal dialysis.
(TIFF)

S6 Fig Estimated probability of transition for a Mixed or Other female aged 62 without diabetes mellitus as primary kidney disease in IMD quintile 3 (moderate deprivation level), commencing KRT on (a) ICHD, (b) PD, (c) HHD or (d) Transplant. IMD, Index of Multiple Deprivation; KRT, kidney replacement therapy; ICHD, in-centre haemodialysis; HHD, home haemodialysis; PD, peritoneal dialysis.
(TIFF)

S1 File Temporal patterns in transition rates, Figs A–K S1 File. IMD, Index of Multiple Deprivation; DM, diabetes mellitus; PKD, primary kidney disease; KRT, kidney replacement therapy; ICHD, in-centre haemodialysis; HHD, home haemodialysis; PD, peritoneal dialysis.
(DOCX)

S1 RECORD Checklist Reporting of Studies Conducted using Observational Routinely-Collected Data (RECORD) guideline. (DOCX)

## Abbreviations

CI: confidence interval
DM: diabetes mellitus
HHD: home haemodialysis
HR: hazard ratio
ICHD: in-centre haemodialysis
IMD: Index of Multiple Deprivation
KRT: kidney replacement therapy
PD: peritoneal dialysis
PH: proportional hazards
PKD: primary kidney disease
RECORD: Reporting of Studies Conducted using Observational Routinely-Collected Data
UKRR: UK Renal Registry
