# Supplementary material for: Health disparities in transitions between kidney replacement therapy modalities and mortality in England: A multistate model using UK Renal Registry data
Source: PLoS Med. 2026 Feb 18;23(2):e1004674. doi: 10.1371/journal.pmed.1004674 (PMC12928565; doi:10.1371/journal.pmed.1004674)
Supplement: S1 Table — KRT: Kidney replacement therapy, IMD, Index of Multiple Deprivation; DM, diabetes mellitus; PKD, primary kidney disease; ICHD, in-centre haemodialysis; HHD, home haemodialysis; PD, peritoneal dialysis. (DOCX) [file pmed.1004674.s001.docx]

**Table S1.** Initial KRT modality, sex, IMD quintile, ethnicity, diabetes mellitus as primary kidney disease (N (%) within each stratum across years) and age (median, IQR) of KRT incident patients,

| **Start year** | **2005** | **2006** | **2007** | **2008** | **2009** | **2010** | **2011** | **2012** | **2013** | **2014** | **2015** | **2016** | **2017** | **2018** | **2019** | **2020** |
| --- | --- | --- | --- | --- | --- | --- | --- | --- | --- | --- | --- | --- | --- | --- | --- | --- |
| **Total Patients** | 4,747 | 5,018 | 5,383 | 5,528 | 5,625 | 5,436 | 5,594 | 5,664 | 5,843 | 6,191 | 6,364 | 6,421 | 6,609 | 6,621 | 6,599 | 5,807 |
| **ICHD** | 2,553 | 3,817 | 4,005 | 4,124 | 4,196 | 3,989 | 4,003 | 4,113 | 4,150 | 4,387 | 4,614 | 4,585 | 4,730 | 4,751 | 4,711 | 4,156 |
|  | 75% | 76% | 74% | 75% | 75% | 73% | 72% | 73% | 71% | 71% | 72% | 71% | 72% | 72% | 71% | 72% |
| **HHD** | 5 (<1%) | 3 (<1%) | 9 (<1%) | 4 (<1%) | 33 (1%) | 5 (<1%) | 4 (<1%) | 4 (<1%) | 3 (<1%) | 16 (<1%) | 6 (<1%) | 7 (<1%) | 11 (<1%) | 13 (<1%) | 13 (<1%) | 8 (<1%) |
| **PD** | 1,033 | 1,017 | 1,102 | 1,091 | 1,039 | 1,031 | 1,176 | 1,127 | 1,162 | 1,266 | 1,248 | 1,317 | 1,290 | 1,315 | 1,360 | 1,332 |
|  | 22% | 20% | 21% | 19% | 19% | 19% | 21% | 20% | 20% | 20% | 20% | 21% | 19% | 20% | 21% | 23% |
| **Transplant** | 156 | 171 | 267 | 309 | 357 | 411 | 411 | 420 | 528 | 522 | 497 | 512 | 578 | 542 | 515 | 311 |
|  | 3% | 3% | 5% | 5% | 6% | 8% | 7% | 7% | 9% | 8% | 8% | 8% | 9% | 8.8% | 8% | 5% |
| **Males** | 2,951 | 3,101 | 3,323 | 3,411 | 3,468 | 3,140 | 3,563 | 3,527 | 3,718 | 3,947 | 4.005 | 4,086 | 4,251 | 4,196 | 4,219 | 3,785 |
|  | 62% | 62% | 62% | 62% | 62% | 63% | 64% | 62% | 64% | 64% | 63% | 64% | 64% | 63% | 64% | 65% |
| **IMD Quintile** |  |  |  |  |  |  |  |  |  |  |  |  |  |  |  |  |
| **1** | 700 | 793 | 864 | 817 | 873 | 817 | 859 | 852 | 885 | 950 | 1,007 | 991 | 981 | 918 | 974 | 961 |
| **(Least deprived)** | 15% | 16% | 16% | 15% | 15% | 15% | 16% | 15% | 15% | 15% | 16% | 15% | 15% | 14% | 14% | 15% |
| **2** | 903 | 893 | 975 | 980 | 1,046 | 990 | 1,012 | 1,000 | 1,024 | 1,098 | 1,097 | 1,119 | 1,130 | 1,120 | 1,122 | 1,030 |
|  | 19% | 18% | 18% | 18% | 19% | 18% | 18% | 18% | 18% | 18% | 17% | 17% | 17% | 17% | 17% | 18% |
| **3** | 942 | 998 | 1,058 | 1,162 | 1,099 | 1,101 | 1,133 | 1,117 | 1,128 | 1,210 | 1,242 | 1,271 | 1,257 | 1,286 | 1,371 | 1,146 |
|  | 20% | 20% | 20% | 21% | 20% | 20% | 20% | 20% | 19% | 20% | 19% | 20% | 19% | 19% | 21% | 20% |
| **4** | 1,080 | 1,128 | 1,171 | 1,216 | 1,278 | 1,200 | 1,229 | 1,305 | 1,353 | 1,395 | 1,434 | 1,435 | 1,556 | 1,561 | 1,507 | 1,314 |
|  | 23% | 23% | 22% | 22% | 23% | 22% | 22% | 23% | 23% | 23% | 22% | 22% | 24% | 24% | 23% | 22% |
| **5** | 1,114 | 1,185 | 1,300 | 1,332 | 1,312 | 1,303 | 1,353 | 1,383 | 1,449 | 1,533 | 1,574 | 1,602 | 1,676 | 1,727 | 1,614 | 1,452 |
| **(Most deprived)** | 24% | 24% | 24% | 24% | 23% | 24% | 24% | 24% | 25% | 25% | 25% | 25% | 25% | 26% | 24% | 25% |
| **Missing** | 8 (<1%) | 21 (<1%) | 15 (<1%) | 21 (<1%) | 17 (<1%) | 25 (<1%) | 8 (<1%) | 7 (<1%) | 4 (<1%) | 5 (<1%) | 11 (<1%) | 3 (<1%) | 9 (<1%) | 9 (<1%) | 11 (<1%) | 5 (<1%) |
| **Ethnicity** |  |  |  |  |  |  |  |  |  |  |  |  |  |  |  |  |
| **Asian** | 476 | 499 | 554 | 596 | 618 | 696 | 699 | 709 | 746 | 756 | 869 | 860 | 889 | 905 | 925 | 808 |
|  | 10% | 10% | 10% | 11% | 11% | 13% | 13% | 13% | 13% | 12% | 14% | 13% | 13% | 14% | 14% | 14% |
| **Black** | 304 | 285 | 369 | 362 | 397 | 347 | 435 | 397 | 437 | 427 | 504 | 500 | 494 | 509 | 488 | 436 |
|  | 6% | 6% | 7% | 7% | 7% | 6% | 8% | 7% | 8% | 7% | 8% | 8% | 8% | 8% | 7% | 8% |
| **Mixed** | 41 | 43 | 52 | 62 | 55 | 57 | 60 | 59 | 62 | 61 | 90 | 96 | 126 | 143 | 95 | 77 |
|  | 1% | 1% | 1% | 1% | 1% | 1% | 1% | 1% | 1% | 1% | 1% | 2% | 2% | 2% | 2% | 1% |
| **Other** | 54 | 38 | 45 | 53 | 76 | 63 | 69 | 87 | 105 | 130 | 90 | 108 | 114 | 111 | 113 | 110 |
|  | 1% | 1% | 1% | 1% | 1% | 1% | 1% | 2% | 2% | 2% | 1% | 2% | 2% | 2% | 2% | 2% |
| **White** | 3,342 | 3,683 | 3,906 | 4,058 | 4,812 | 4,204 | 4,260 | 4,346 | 4,390 | 4,651 | 4,678 | 4,653 | 4,761 | 4,637 | 4,500 | 3,914 |
|  | 70% | 73% | 73% | 73% | 74% | 77% | 76% | 77% | 75% | 75% | 74% | 72% | 72% | 70% | 68% | 68% |
| **Missing** | 530 | 470 | 457 | 397 | 297 | 69 | 71 | 66 | 103 | 166 | 134 | 204 | 225 | 316 | 478 | 463 |
|  | 11% | 9% | 8% | 7% | 5% | 1% | 1% | 1% | 2% | 3% | 2% | 3% | 3% | 5% | 7% | 8% |
| **Age** | 65 | 65 | 64 | 64 | 64 | 65 | 65 | 64 | 64 | 65 | 65 | 64 | 64 | 64 | 64 | 64 |
|  | (50-74) | (51-74) | (50-74) | (50-74) | (51 -75) | (51-75) | (51-75) | (51-75) | (51-75) | (52-75) | (51-75) | (51-74) | (51-74) | (51-74) | (52-74) | (52-74) |
| **No DM as PKD** | 3,571 | 3,705 | 4,016 | 4,071 | 4,119 | 4,013 | 4,063 | 4,130 | 4,165 | 4,495 | 4,505 | 4,470 | 4,328 | 4,362 | 4,073 | 3,513 |
|  | 75% | 74% | 75% | 74% | 73% | 74% | 73% | 73% | 71% | 73% | 71% | 70% | 65% | 66% | 62% | 61% |
| **DM as PKD** | 988 | 1,090 | 1,178 | 1,226 | 1,322 | 1,235 | 1,273 | 1,390 | 1,426 | 1,569 | 1,656 | 1,695 | 1,762 | 1,856 | 1,775 | 1,593 |
|  | 20% | 22% | 22% | 23% | 24% | 23% | 23% | 25% | 24% | 25% | 26% | 26% | 27% | 28% | 27% | 27% |
| **Missing** | 232 | 223 | 189 | 191 | 184 | 188 | 258 | 144 | 252 | 127 | 204 | 204 | 519 | 403 | 751 | 701 |
|  | 5% | 4% | 3% | 3% | 3% | 3% | 4% | 2% | 4% | 2% | 3% | 3% | 8% | 6% | 11% | 12% |

IMD: Index of multiple deprivation. DM: Diabetes mellitus, PKD: Primary kidney disease
