# Supplementary material for: Health disparities in transitions between kidney replacement therapy modalities and mortality in England: A multistate model using UK Renal Registry data
Source: PLoS Med. 2026 Feb 18;23(2):e1004674. doi: 10.1371/journal.pmed.1004674 (PMC12928565; doi:10.1371/journal.pmed.1004674)
Supplement: S2 Table — a There are no restrictions in the number of modality changes a patient can undergo; therefore, patients may experience multiple modality transition pairings. b Transition not included in the multistate model due to very low frequency. ICHD, in-centre haemodialysis; HHD, home haemodialysis; PD, peritoneal dialysis. (DOCX) [file pmed.1004674.s002.docx]

**Table S2.** Frequency of transition between treatment modalities ^a^ or to death, with previous modality cross-tabulated against current modality at successive observation times

| **To**  **From** | **ICHD** | **HHD** | **PD** | **Transplantation** | **Death** | **Total** |
| --- | --- | --- | --- | --- | --- | --- |
| **ICHD** |  | 3,764 (6%) | 7,061 (12%) | 14,398 (23%) | 36,308 (59%) | 61,531 |
| **HHD** | 2,010 (63%) |  | 32^b^ (1%) | 780 (24%) | 392 (12%) | 3,214 |
| **PD** | 12,580 (56%) | 68^b^ (<1%) |  | 5,730 (25%) | 4,173 (19%) | 22,551 |
| **Transplantation** | 3,202 (53%) | 32^b^ (<1%) | 433 (7%) |  | 2,347 (39%) | 6,014 |

^a^ There are no restrictions in the number of modality changes a patient can undergo; therefore, patients may experience multiple modality transition pairings.

^b^ Transition not included in the multistate model due to very low frequency.
