# Supplementary material for: Health disparities in transitions between kidney replacement therapy modalities and mortality in England: A multistate model using UK Renal Registry data
Source: PLoS Med. 2026 Feb 18;23(2):e1004674. doi: 10.1371/journal.pmed.1004674 (PMC12928565; doi:10.1371/journal.pmed.1004674)
Supplement: S3 Table — a Estimated for a White male aged 62 with diabetes mellitus not as primary kidney disease in IMD quintile 3 (moderate area-level deprivation). ICHD, in-centre haemodialysis; HHD, home haemodialysis; PD, peritoneal dialysis; IMD, Index of Multiple Deprivation. (DOCX) [file pmed.1004674.s003.docx]

**Table S3.** Estimated median time spent in each state, in days (inter-quartile range), before transitioning to the next state^a^ and overall median time spent in same modality

| **To**  **From** | **ICHD** | **PD** | **HHD** | **Transplant** | **Death** | **Patients (n=28,279) who remained in same modality (%)** | **Time spent in same modality** |
| --- | --- | --- | --- | --- | --- | --- | --- |
| **ICHD** |  | 217 (28-714) | 408 (93-1222) | 941 (342-2011) | 1134 (455-2251) | 19,795 (70%) | 1075 (503-2057) |
| **PD** | 417 (135-962) |  |  | 660 (277-1278) | 770 (361-1530) | 24 (<1%) | 569 (246-1100) |
| **HHD** | 329 (95-894) |  |  | 674 (284-1434) | 851 (376-1624) | 2,975 (11%) | 659 (363-948) |
| **Transplant** | 1240 (358-2611) | 1237 (350-2700) |  |  | 2080 (970-3452) | 5,485 (19%) | 2087 (1080-3363) |

^a^ Estimated for a White male aged 62 with diabetes mellitus not as primary kidney disease in IMD quintile 3 (moderate area-level deprivation)
