# Supplementary figures and images for: Health disparities in transitions between kidney replacement therapy modalities and mortality in England: A multistate model using UK Renal Registry data
Source: PLoS Med. 2026 Feb 18;23(2):e1004674. doi: 10.1371/journal.pmed.1004674 (PMC12928565; doi:10.1371/journal.pmed.1004674)

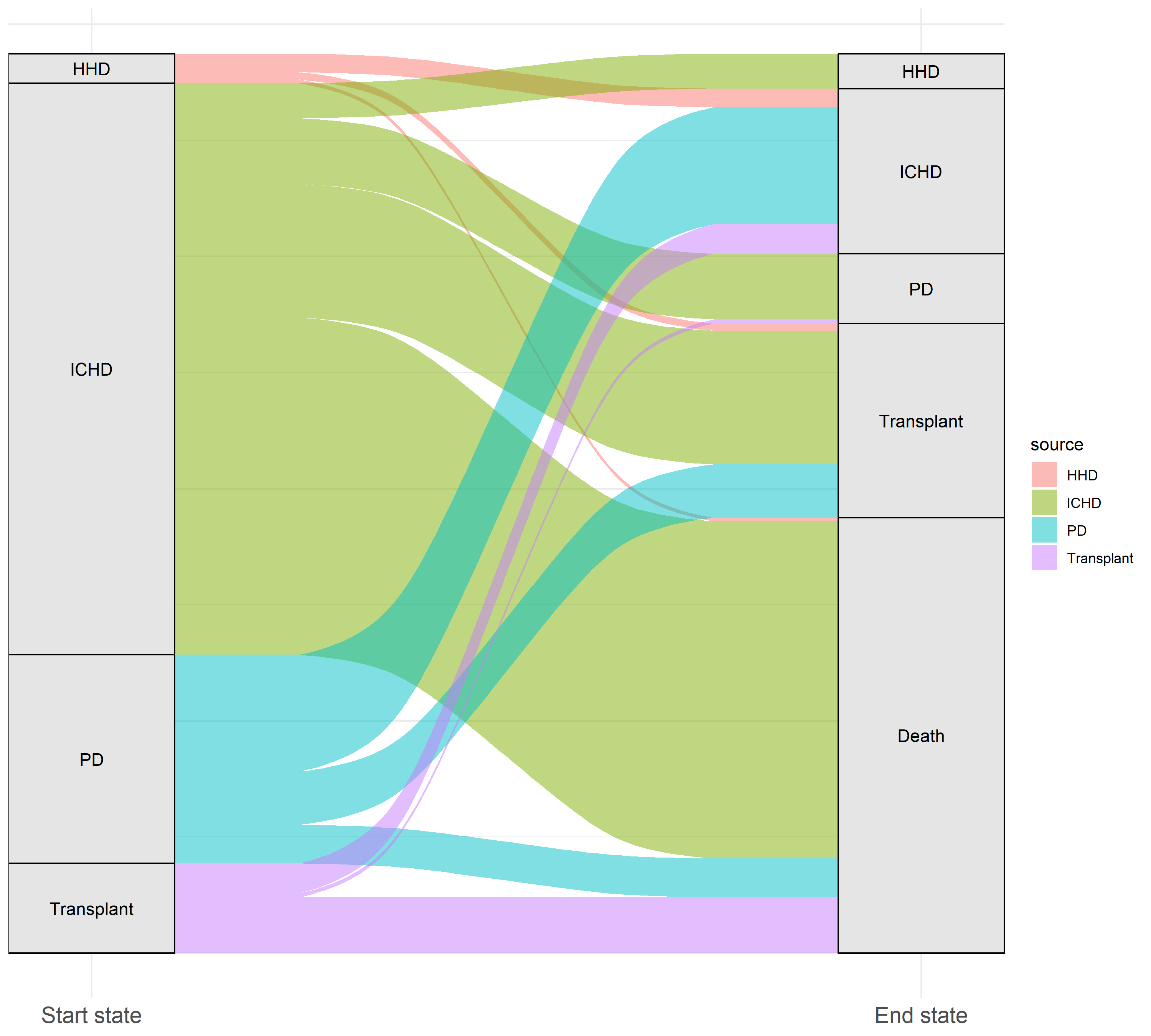

Supplement: S4 Fig — The width of each flow is proportional to the number of patients transitioning between states. ICHD, in-centre haemodialysis; HHD, home haemodialysis; PD, peritoneal dialysis. (TIFF) [file pmed.1004674.s007.tiff]

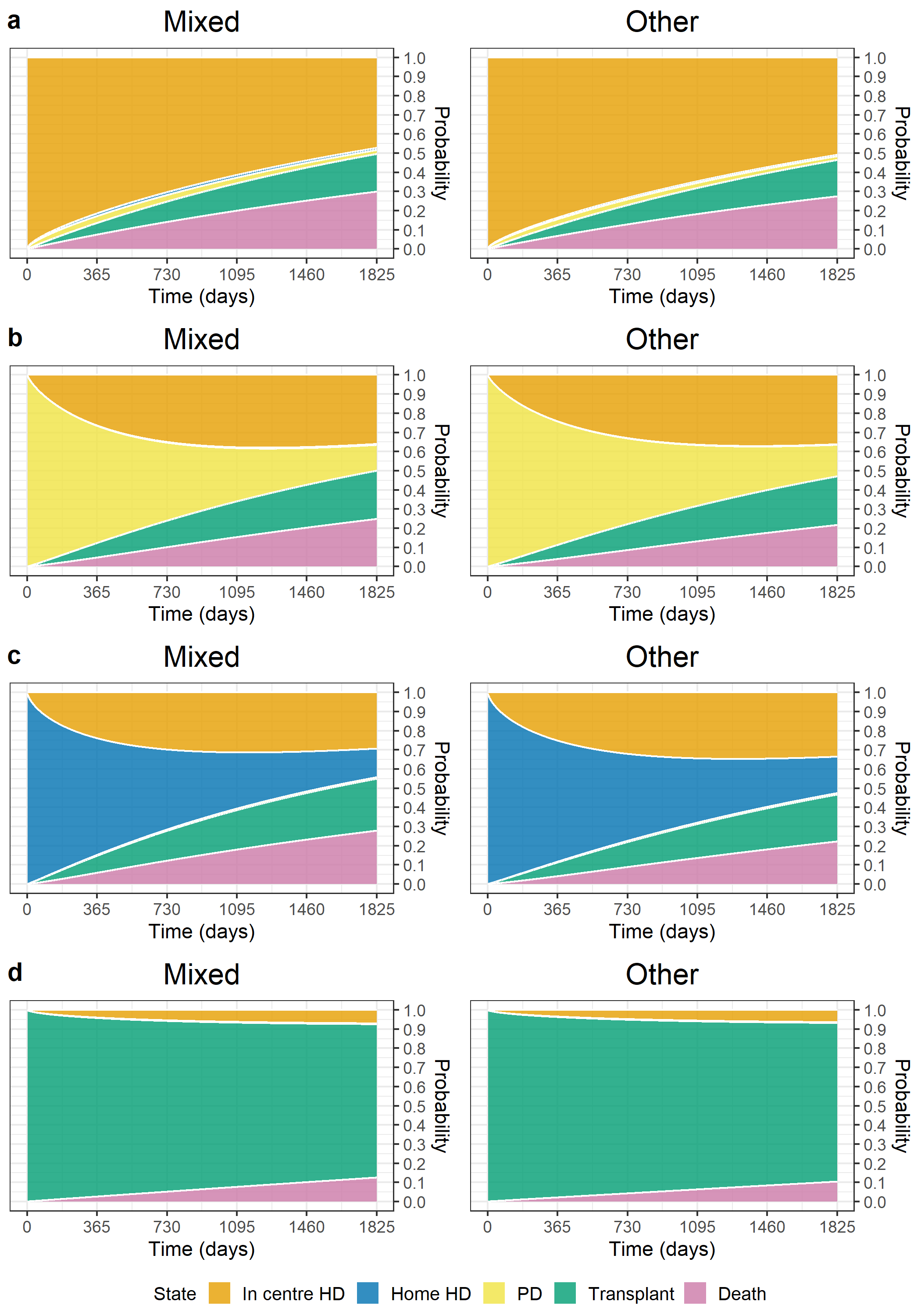

Supplement: S5 Fig — IMD, Index of Multiple Deprivation; KRT, kidney replacement therapy; ICHD, in-centre haemodialysis; HHD, home haemodialysis; PD, peritoneal dialysis. (TIFF) [file pmed.1004674.s008.tiff]

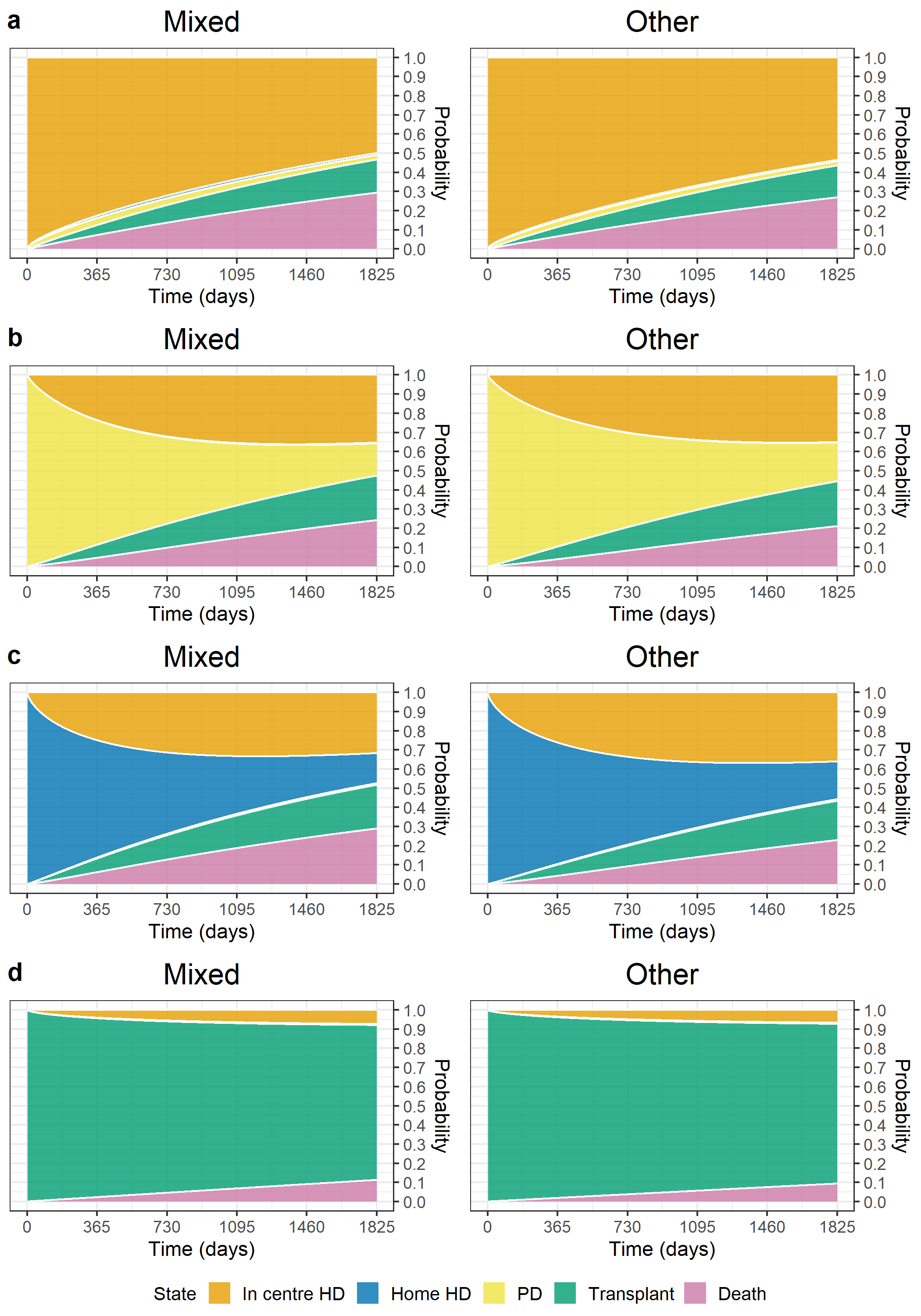

Supplement: S6 Fig — IMD, Index of Multiple Deprivation; KRT, kidney replacement therapy; ICHD, in-centre haemodialysis; HHD, home haemodialysis; PD, peritoneal dialysis. (TIFF) [file pmed.1004674.s009.tiff]
