# Supplementary material for: Health disparities in transitions between kidney replacement therapy modalities and mortality in England: A multistate model using UK Renal Registry data
Source: PLoS Med. 2026 Feb 18;23(2):e1004674. doi: 10.1371/journal.pmed.1004674 (PMC12928565; doi:10.1371/journal.pmed.1004674)

Temporal patterns in transition rates: Hazard ratios for transitions between treatment modalities and mortality in analyses stratified by patient characterisitcs and period, 2005-2009, 2010-2014, 2015-2020 and overall (2005-2020)

Note: No symbols or bolding are used to denote statistical significance, as confidence intervals are unadjusted for multiple comparisons; readers are encouraged to consider overall patterns rather than isolated differences

Figure A. Hazard ratios for transitions between treatment modalities and mortality for Asian (vs White) patients in analyses stratified by period, 2005-2009, 2010-2014, 2015-2020 and overall (2005-2020)


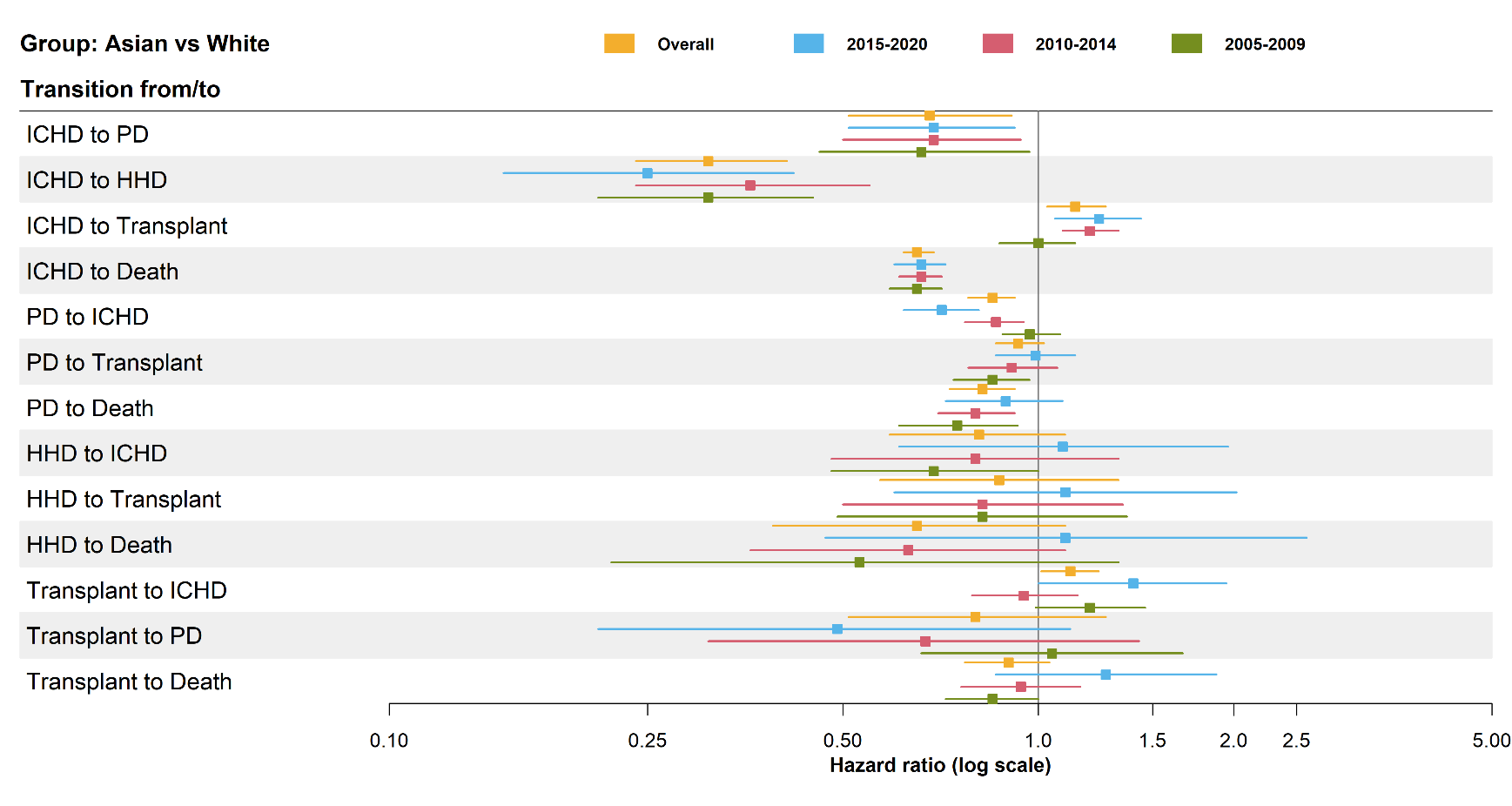


[Figure B. Hazard ratios for transitions between treatment modalities and mortality for Black (vs White) patients in analysis stratified by period, 2005-2009, 2010-2014, 2015-2020, and overall (2005-2020)](#_Toc209536251)


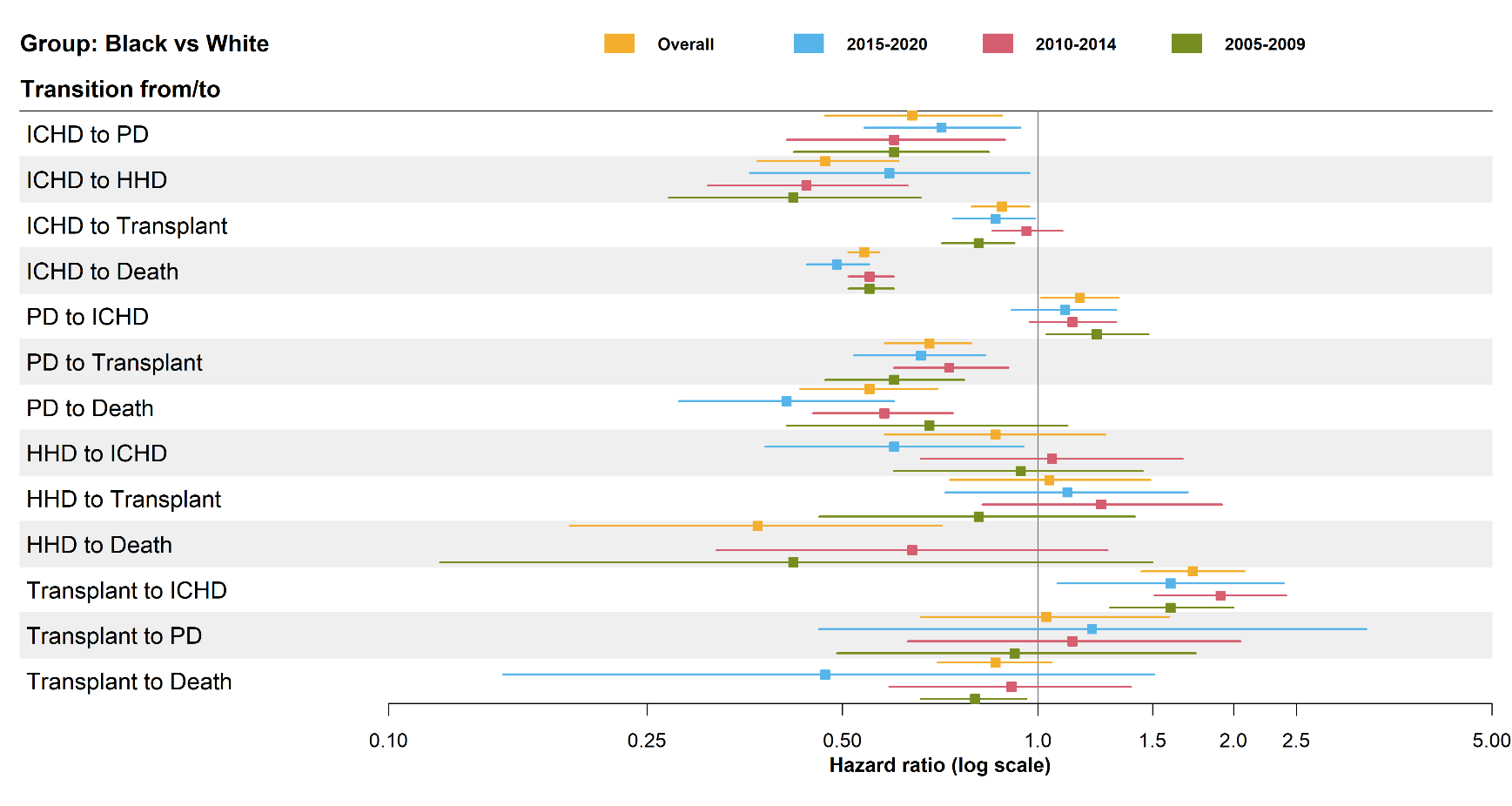


[Figure C. Hazard ratios for transitions between treatment modalities and mortality for Mixed (vs White) patients in analysis stratified by period, 2005-2009, 2010-2014, 2015-2020 and overall (2005-2020)](#_Toc209536252)
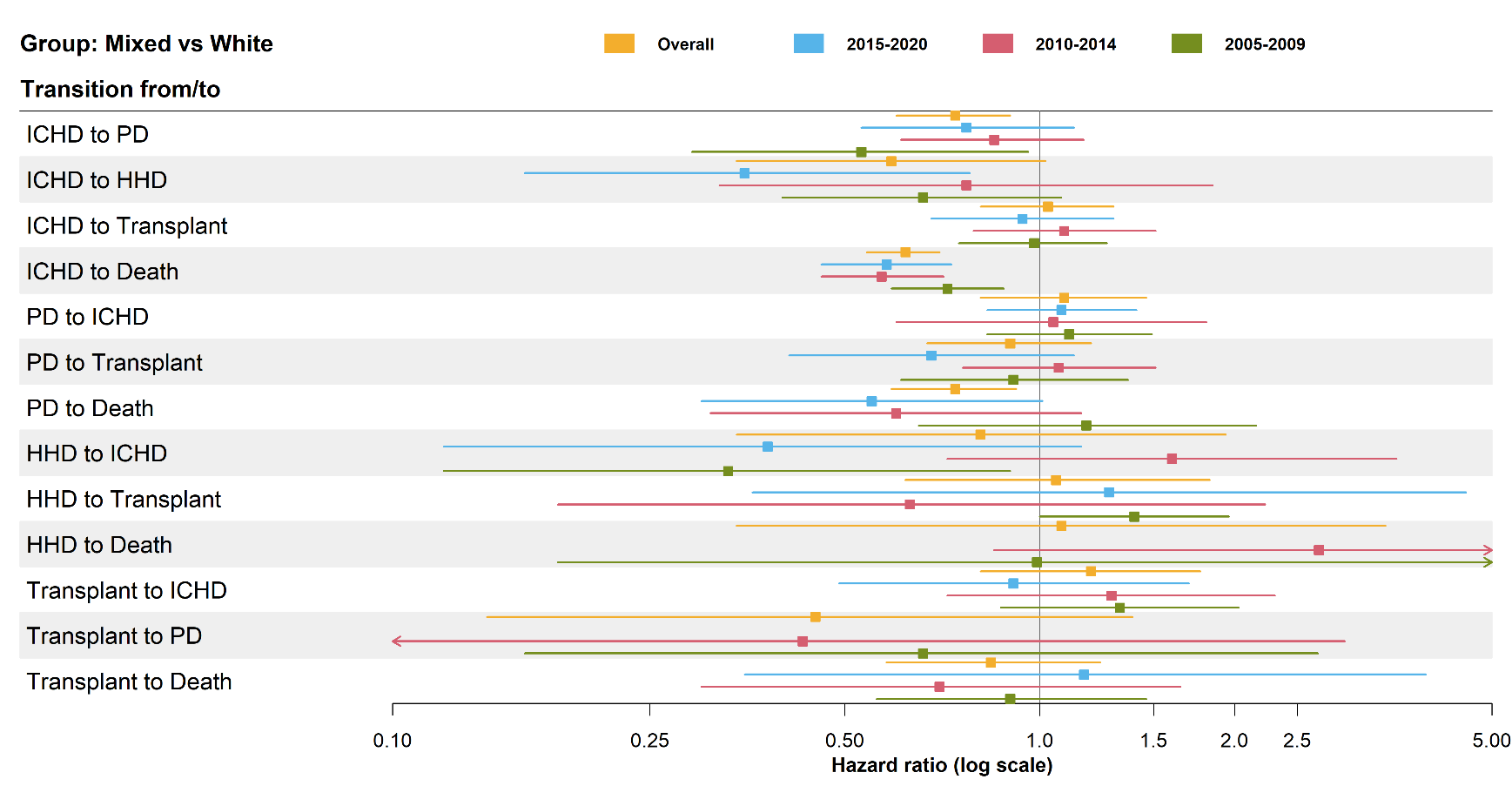


[Figure D. Hazard ratios for transitions between treatment modalities and mortality for Other (vs White) ethnic group in analyses stratified by period, 2005-2009, 2010-2014, 2015-2020, and overall (2005-2020)](#_Toc209536253)
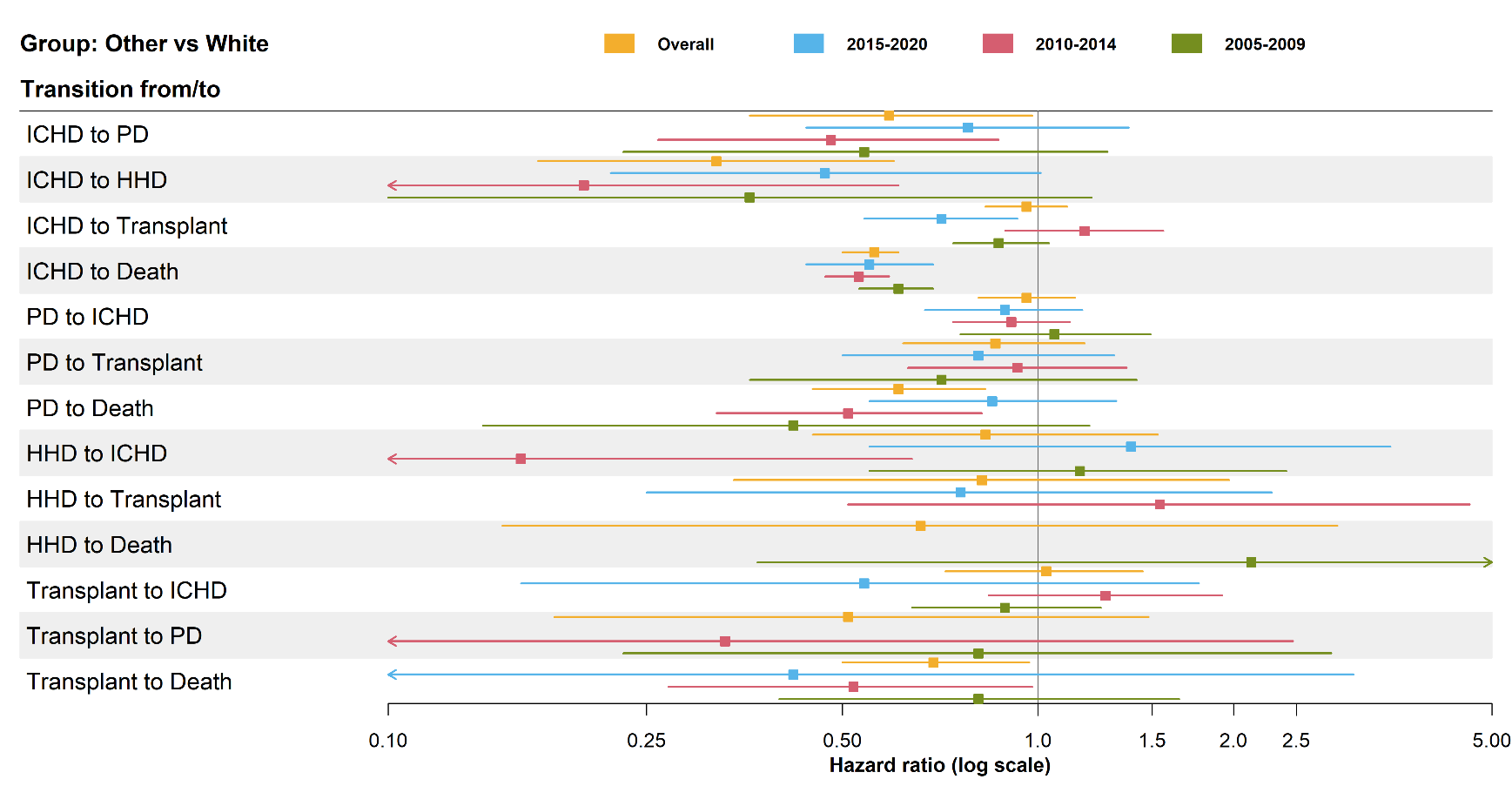


[Figure E. Hazard ratios for transitions between treatment modalities and mortality for IMD quintile 2 vs IMD quintile 1 (Least deprived) group in analysis stratified by period, 2005-2009, 2010-2014, 2015-2020 and overall (2005-2020)](#_Toc209536254)


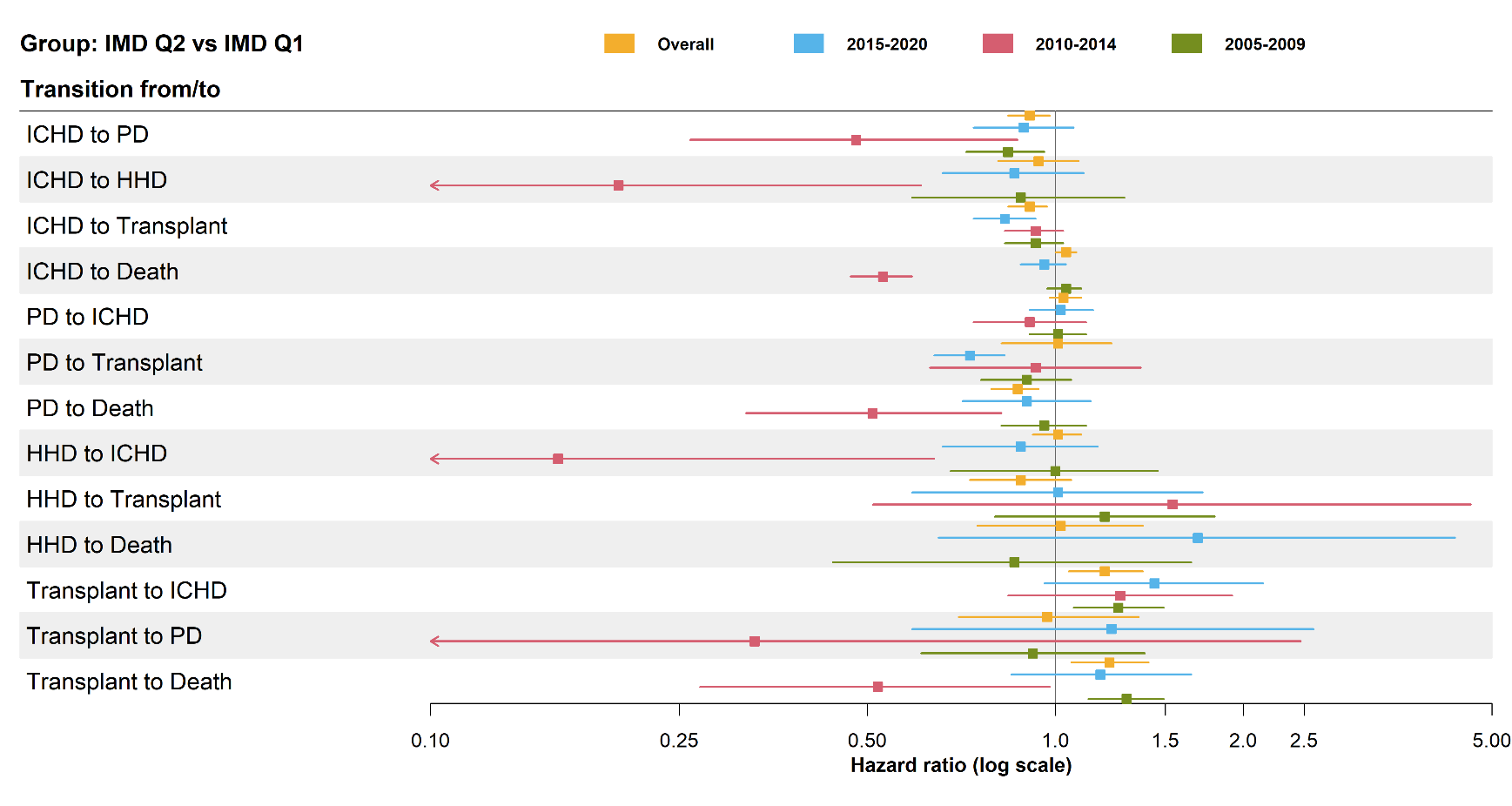


[Figure F. Hazard ratios for transitions between treatment modalities and mortality for IMD quintile 3 vs IMD quintile 1 (Least deprived) group in analyses stratified by period, 2005-2009, 2010-2014, 2015-2020 and overall (2005-2020)](#_Toc209536255)


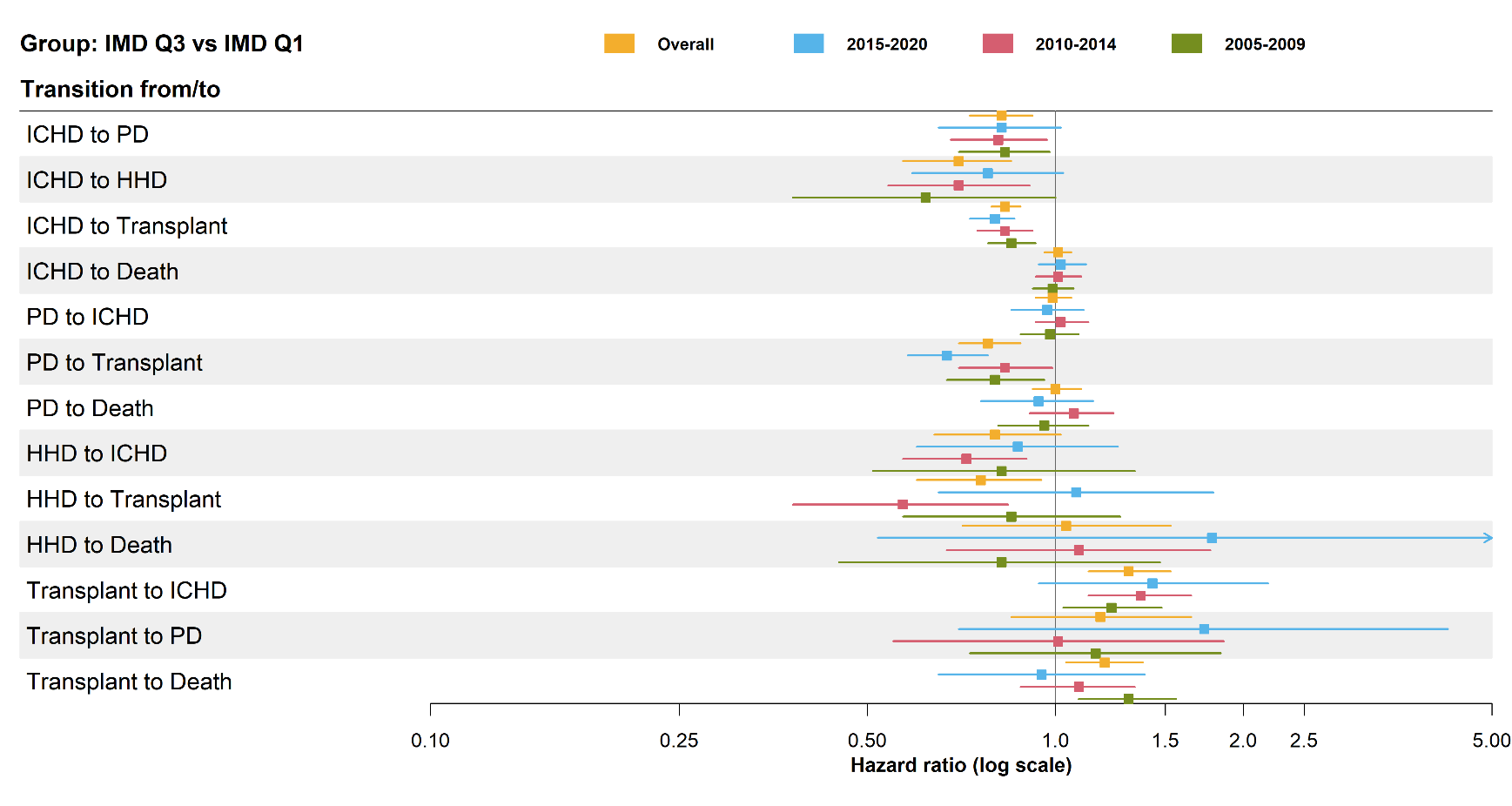


[Figure G. Hazard ratios for transitions between treatment modalities and mortality for IMD quintile 4 vs IMD quintile 1 (Least deprived) group in analyses stratified by period, 2005-2009, 2010-2014, 2015-2020 and overall (2005-2020)](#_Toc209536256)


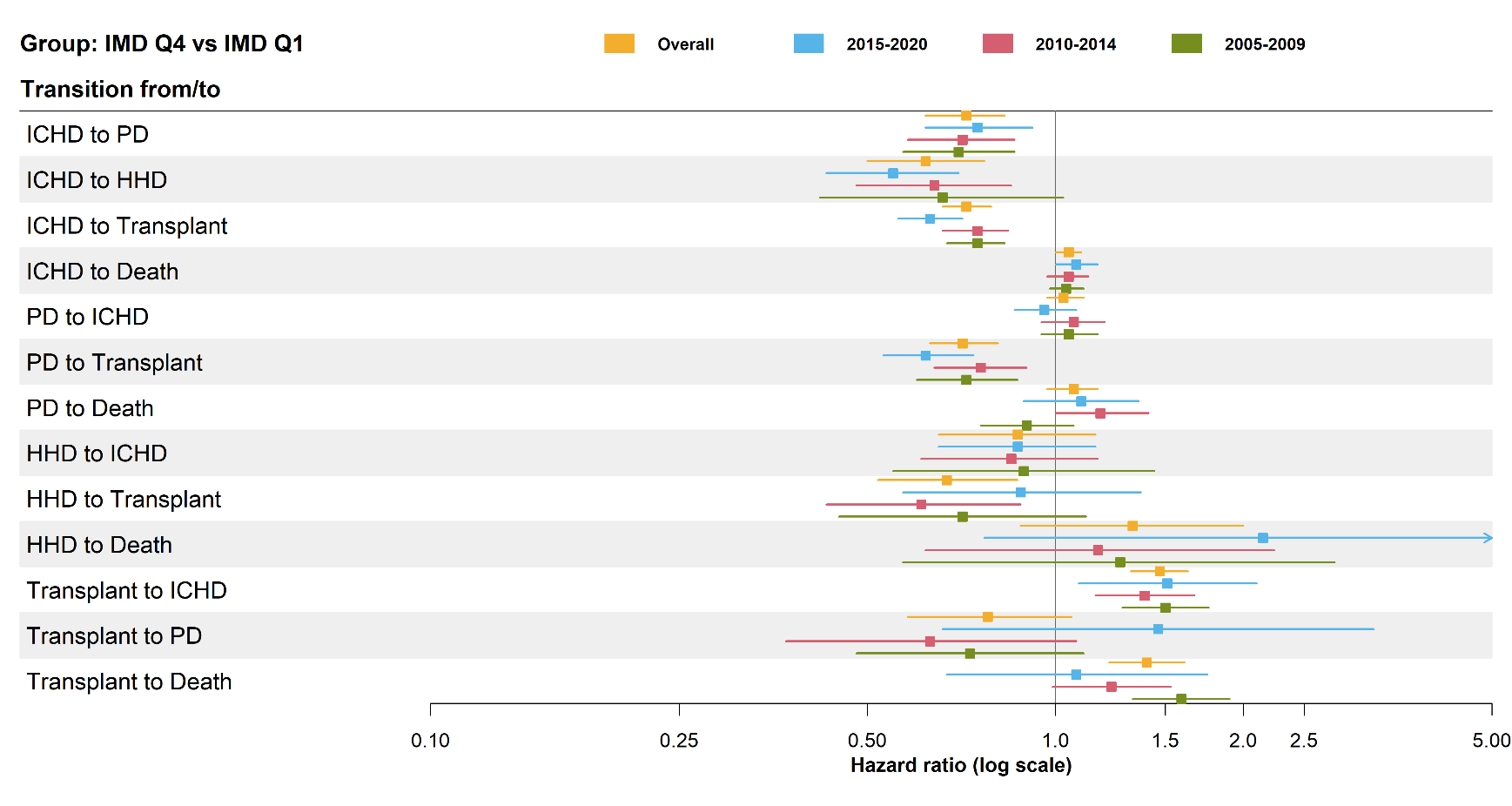


[Figure H. Hazard ratios for transitions between treatment modalities and mortality for IMD quintile 5 vs IMD quintile 1 (Least deprived) group in analyses stratified by period, 2005-2009, 2010-2014, 2015-2020 and overall (2005-2020)](#_Toc209536257)


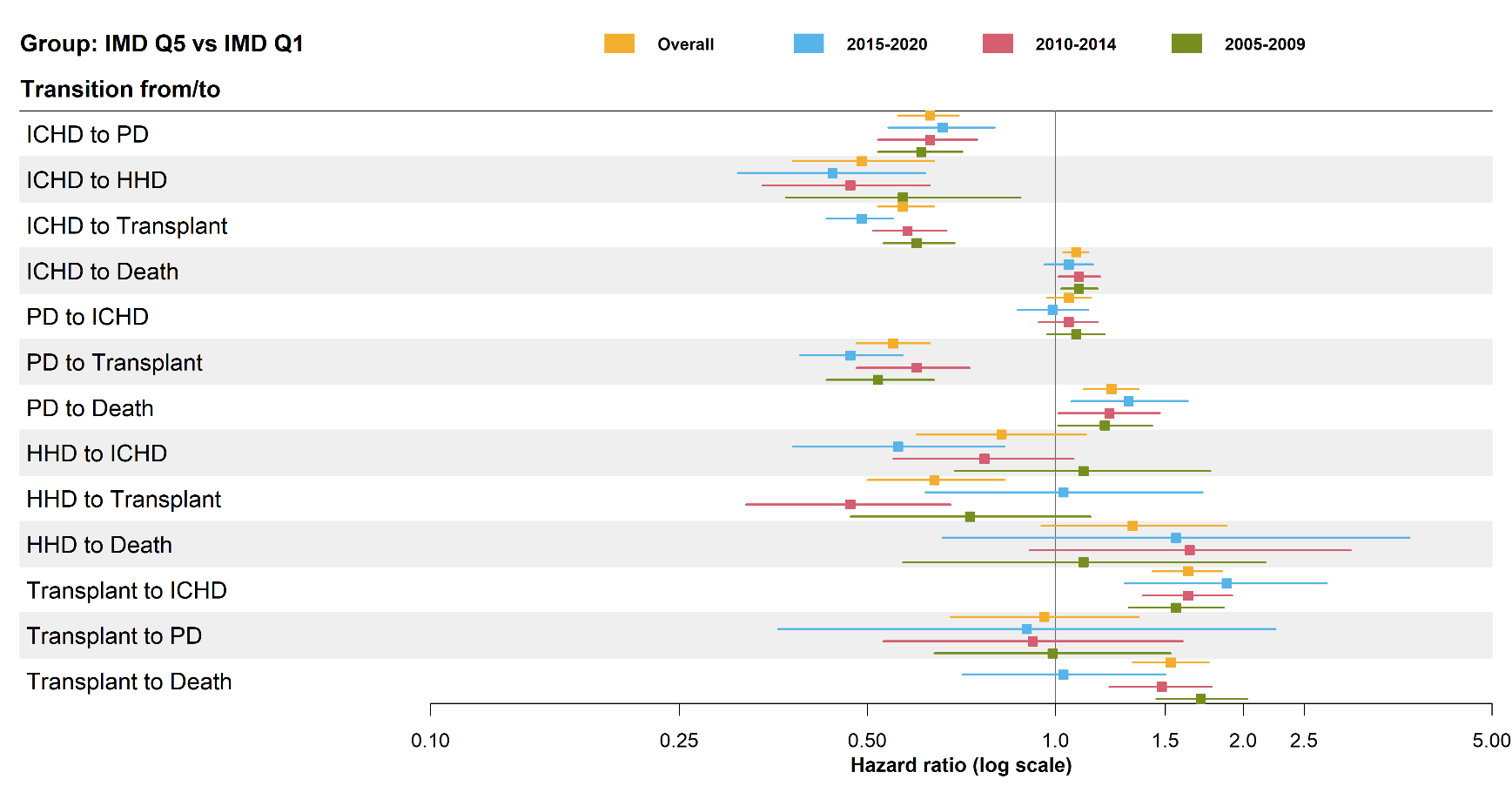


[**Figure I. Hazard ratios for transitions between treatment modalities and mortality for female (vs male) patients in analyses stratified by period, 2005-2009, 2010-2014, 2015-2020, and overall (2005-2020)**](#_Toc209536248)
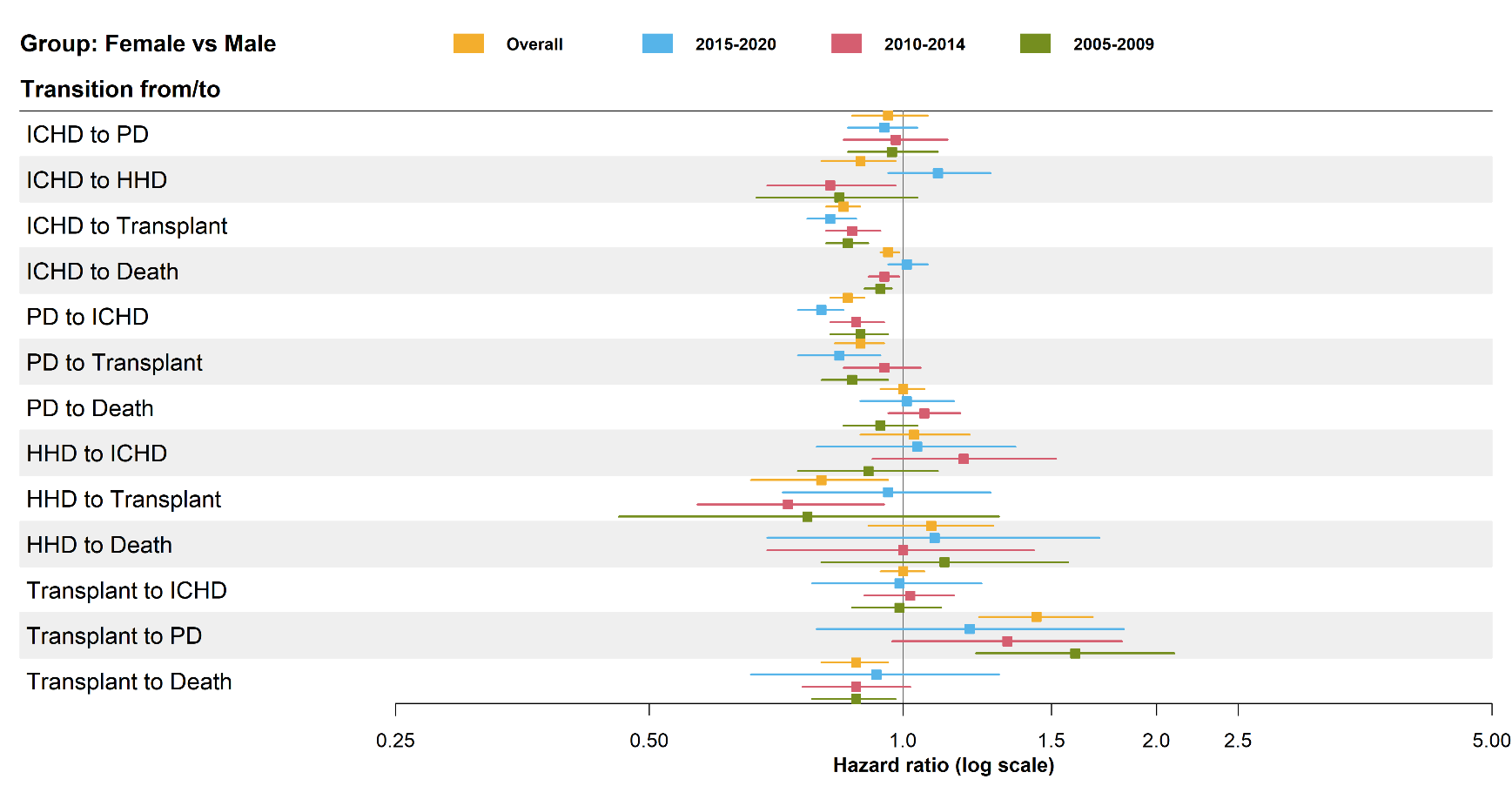


[Figure J. Hazard ratios for transitions between treatment modalities and mortality for each incremental year of age in analyses stratified by period, 2005-2009, 2010-2014, 2015-2020 and overall (2005-2020)](#_Toc209536249)


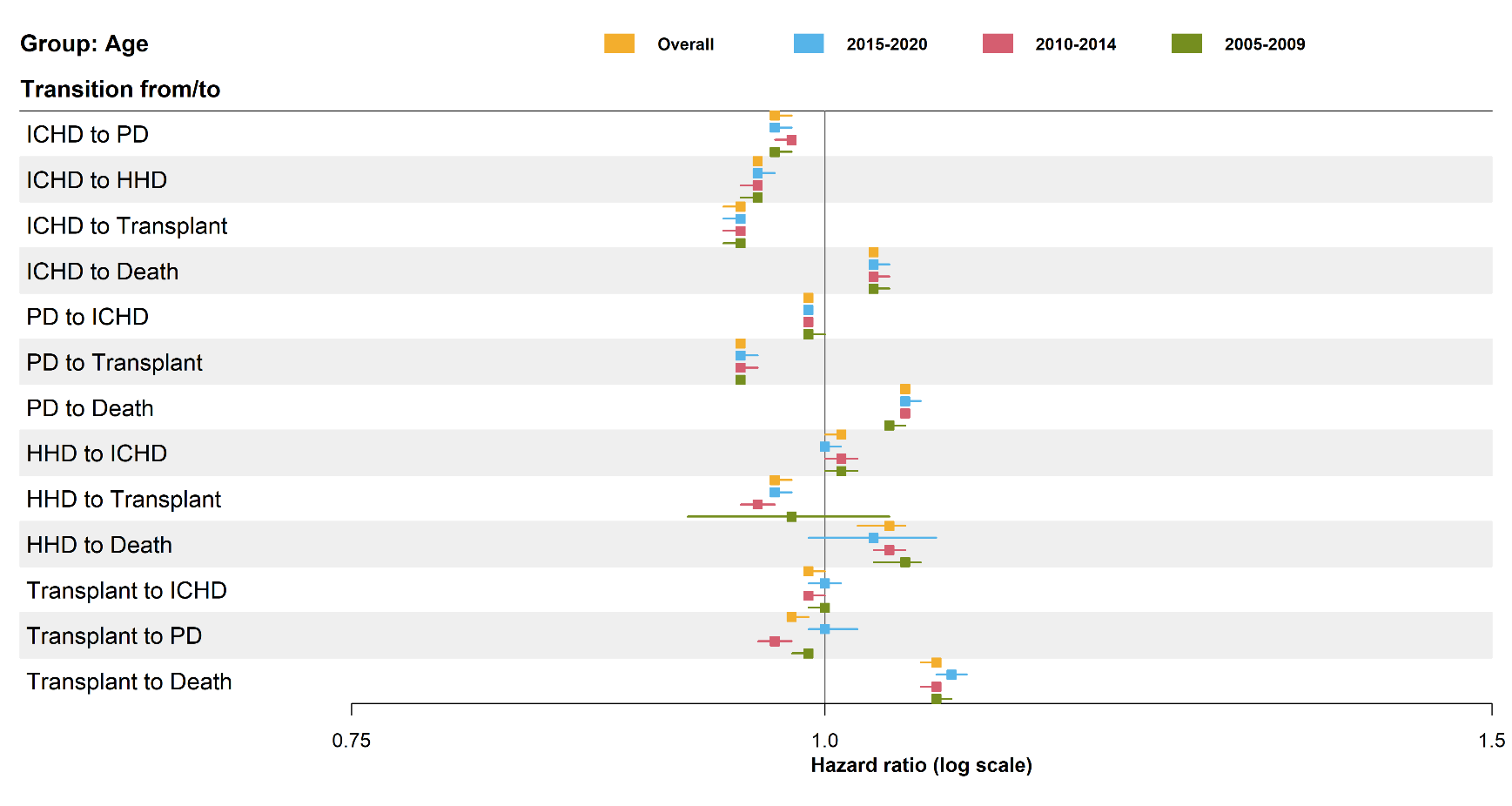


[Figure K. Hazard ratios for transitions between treatment modalities and mortality for patients who had diabetes as PKD vs this who did not stratified by period, 2005-2009, 2010-2014, 2015-2020 and overall (2005-2020)](#_Toc209536258)


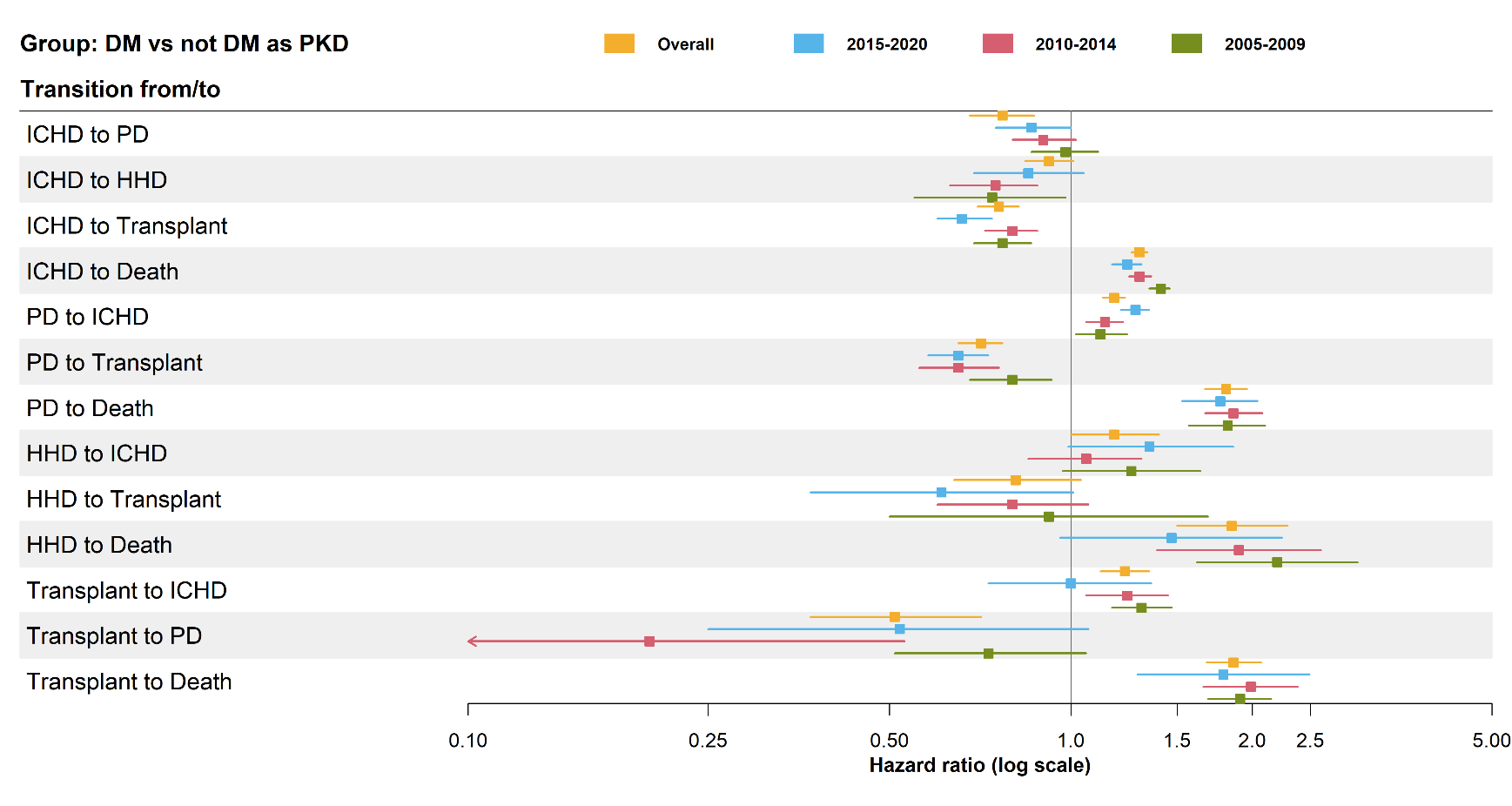

Supplement: S1 File — IMD, Index of Multiple Deprivation; DM, diabetes mellitus; PKD, primary kidney disease; KRT, kidney replacement therapy; ICHD, in-centre haemodialysis; HHD, home haemodialysis; PD, peritoneal dialysis. (DOCX) [file pmed.1004674.s010.docx]
